# Macrocyclic Triazolopeptoids: A Promising Class of Extended Cyclic Peptoids

**DOI:** 10.1021/acs.orglett.2c03062

**Published:** 2022-10-12

**Authors:** Alicja M. Araszczuk, Assunta D’Amato, Rosaria Schettini, Chiara Costabile, Giorgio Della Sala, Giovanni Pierri, Consiglia Tedesco, Francesco De Riccardis, Irene Izzo

**Affiliations:** Department of Chemistry and Biology “A. Zambelli”, University of Salerno, via Giovanni Paolo II, 132, Fisciano, SA 84084, Italy

## Abstract

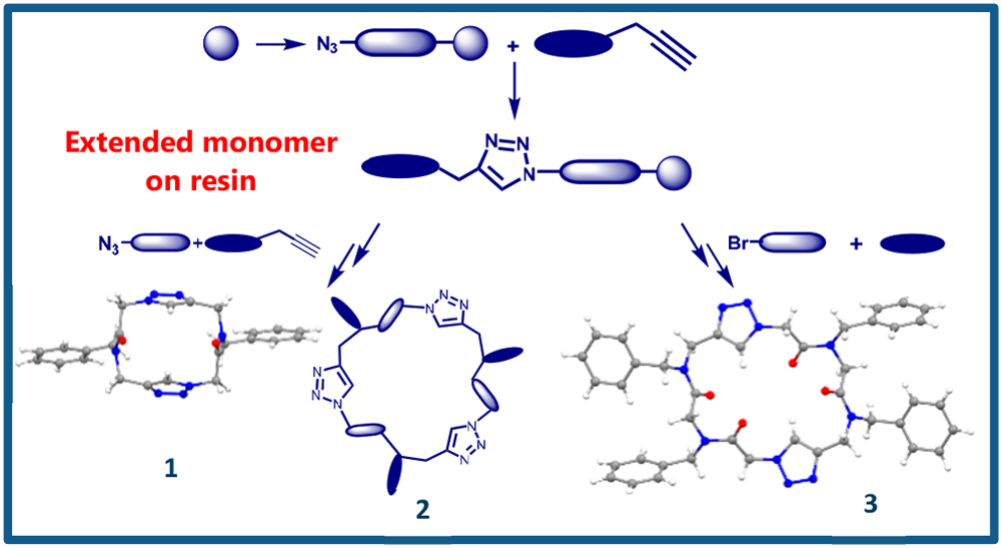

Head-to-tail cyclization of linear oligoamides containing 4-benzylaminomethyl-1*H*-1,2,3-triazol-1-yl acetic acid monomers afforded a novel class of “extended macrocyclic peptoids”. The identification of the conformation in solution for a cyclodimer and the X-ray crystal structure of a cyclic tetraamide are reported.

The intrinsic attitude of macrocyclic systems toward recognition and complexation makes them formidable tools in supramolecular chemistry. New macrocyclic derivatives are continuously reported and investigated, confirming that every class has its own unique features and finds application in different fields.^1^ However, despite the multitude of the structures of known hosts (i.e., cyclodextrins, calixarenes, resorcinarenes, cucurbiturils, etc.), scant synthetic effort has been devoted to sequence-defined oligomerization processes.^2^ A modular accretion of bifunctional monomers is instead adopted for the synthesis of cyclic peptoids (cyclic oligomers of N-substituted glycines),^3,4^ a growing class of biomimetic compounds with enormous potential in catalysis,^5^ in material chemistry,^6^ as bioactive agents,^7,8^ and as precursors of azamacrocycles.^9^ The versatility of their synthetic method allows ample structural flexibility and facile introduction of rigid aromatic spacers into the peptoid oligoamide backbone, producing the valuable “extended peptoids”,^10,11^ with excellent conformational properties^12,13^ (despite the absence of intramolecular H-bonding) and metal chelating abilities.^14^

The richness of the heteroaromatic spacers available (furane, oxazoles, thiazole, etc.) can further expand the chemical space of the N-alkylated aromatic cyclic peptoids, and new studies are increasingly emerging in the past several years.^15,16^ Among the heteroaromatic spacer triazoles are ideal candidates. These intriguing heterocyclic compounds have broad use in the field of peptidomimetics.^17^ Because of their physicochemical resemblance to peptide bonds due to planarity and strong dipole moment, they have been used as amide bond isosteres in peptide science.^18^ In particular, their 1,4-disubstituted 1,2,3-regioisomers, built through the straightforward Cu(I)-catalyzed azide–alkyne cycloaddition (CuAAC) reaction, have already been used in peptoid chemistry for macrocyclization^19−21^ and conjugation with azido-functionalized bioactive compounds.^22−27^ Moreover, triazoles display various supramolecular interactions,^28^ such as hydrogen and halogen bond formation and metal coordination, which can reinforce the well-known complexation abilities of cyclic peptoids.^29^

In this work, we report the synthesis and characterization of dimer **1**, trimer **2**, and tetraoligamide **3** (Figure 1), as first members of cyclic triazolopeptoids, and disclose the structural attitudes of this new class of macrocycles.

**Figure 1:**
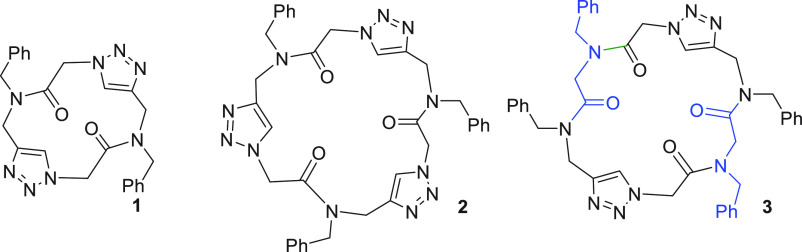
Structures of new macrocyclic triazolopeptoids **1–3** reported herein.

To take advantage of the submonomer-based approach^30^ to the solid phase, azidoacetic acid **4**(31) and *N*-benzylpropargyl amine **5**(32) were prepared in solution in high yields. In particular, the reductive amination,^32^ yielding propargyl-armed **5**, shows the potential of the synthetic approach, which, in principle, can lead to differently decorated macrocycles.

Scheme 1 summarizes the synthetic steps toward triazolopeptoids **1–3**. After the first submonomer **4** had been loaded, the (3+2) Cu(I)-catalyzed cycloaddition reaction smoothly afforded the first “extended” monomer on resin. This was acylated with azidoacetic acid **4** or bromoacetic acid **6**, depending on the requested target(s). Iteration of cycloaddition/acylation steps afforded in one case (after the cleavage from the resin with HFIP 20% in DCM) the desired linear precursors **8** and **9** in quantitative yield. On the contrary, *N*-benzyl glycine addition and iteration of the synthetic steps produced linear precursor **10** in quantitative yield. HATU-induced macrocyclization under high-dilution conditions gave targets **1–3** in 34%, 28%, and 24% yields, respectively. ^1^H and ^13^C NMR spectral analysis of cyclodimer **1** showed the presence of a conformationally stable symmetric compound (Figure 2).^33^ The presence of tertiary amides in cyclic oligomers often induces the coexistence of several conformations in slow equilibrium on the NMR time scale. While smaller cyclic trimeric and tetrameric peptoids appear as single conformers (on the NMR time scale), larger oligomeric macrocycles (e.g., hexamers) show multiple conformations for the limited n → π* orbital contacts^4,33^ and weaker geometric constraints. To understand the structure of the observed conformer **1** and the interactions stabilizing its backbone, in-depth computational studies were performed.

**Scheme 1:**
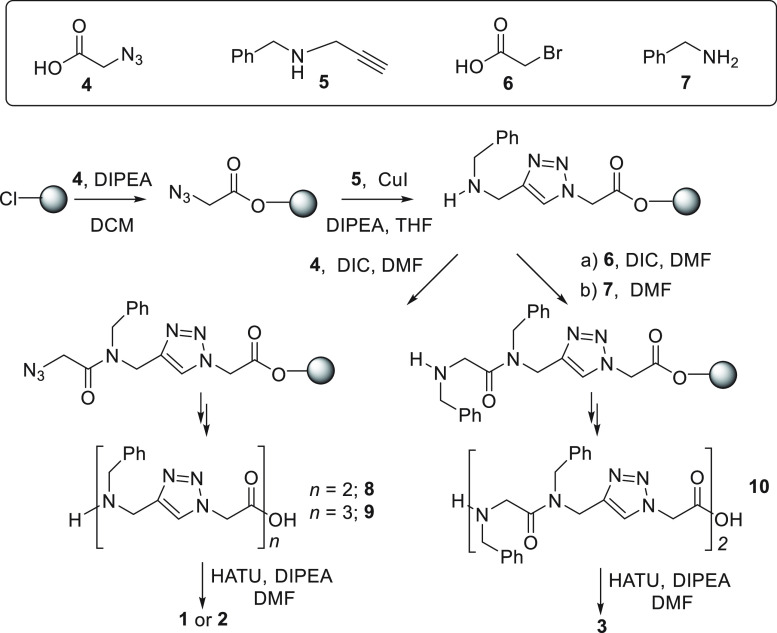
Synthetic Procedure for Linear Oligomeric Precursors **8–10**, Prepared on 2-Chlorotrityl Chloride Resin 1% DVB, and Macrocyclic Targets **1–3**

**Figure 2:**
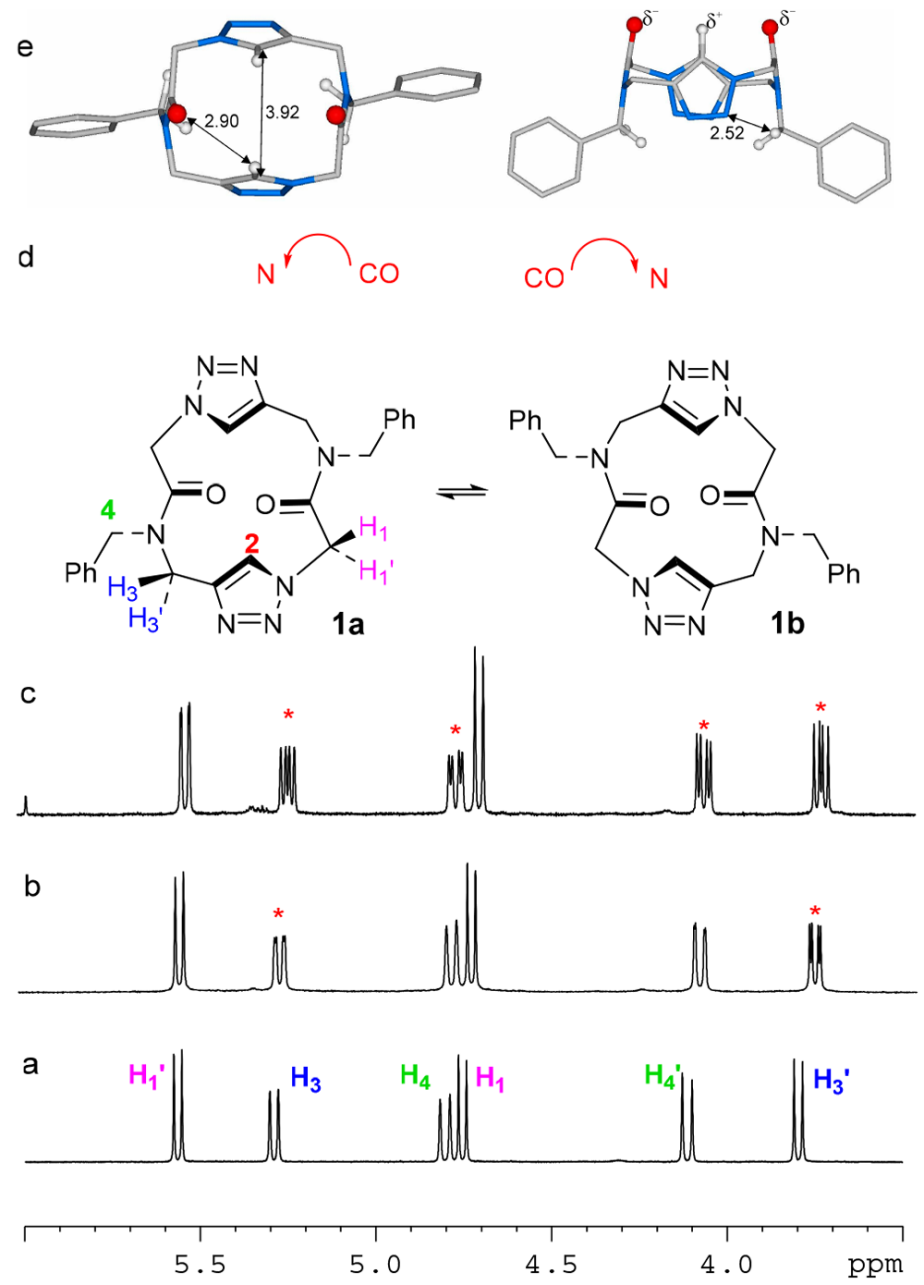
Spectral and theoretical data for cyclodimer **1**. (a) Expansion of its ^1^H NMR spectrum (600 MHz, CDCl_3_, 298 K, 8.0 mM solution). (b and c) Quantitative stepwise addition of 1.0 and 3.0 equiv of Pirkle’s alcohol to conformational enantiomers **1a** and **1b**, respectively. Red asterisks denote split signals. (d) Conformational enantiomers of cyclodimer **1**. (e) Minimum energy conformation of dimer **1**, top (left) and side (right) views. Enantiomer **1a** is reported in the illustration.

According to the 2-fold symmetry revealed by the NMR spectra, cyclodimer **1** could adopt, in principle, a *C*_2_- or *S*_2_-symmetric all-*trans* or all-*cis* conformation (see Figure S12 and Table S1 for structures and energies, respectively). DFT calculations at the BP86/TZVP level identified the all-*trans C*_2_-symmetric conformer (Figure 2e) as the most stable structure (the *S*_2_-symmetric all-*trans* and the *C*_2_- and *S*_2_-symmetric all-*cis* conformers showed Δ*G* values that were 4.1, 6.5, and 5.7 kcal/mol higher, respectively, than that of the all-*trans C*_2_-symmetric conformer).

In the “square” all-*trans C*_2_-symmetric conformation, the two triazole rings act as H-bond acceptors of the CH_2_ benzyl side chains and as carbonyl H-bond donors (Figure 2e). The latter interaction justified the CH triazole ^1^H NMR downfield shift (7.84 ppm).^34^ The assignment was confirmed by a ROESY experiment (see the Supporting Information) that showed, on one hand, cross-peaks between triazole proton H_2_ at 7.84 ppm and β protons H_3_ and H_1_ and, on the other, between the *ortho* protons of aromatic rings and α protons H_1_′ and H_3_′ (Figure 2). Interestingly, the backbone conformation of the most stable conformer closely resembles [root-mean-square deviation 0.163 Å (Figure S15)] that observed by Ghadiri and co-workers^18b^ for the analogous bis-triazole cyclic peptide (X-ray crystal structure, CSD code SURWUY).

The *C*_2_-symmetry implied the presence of two enantiomorphous conformational isomers^35^**1a** and **1b** (Figure 2d), which could be interconverted by simultaneous inversion of the two amide bonds and triazole rings.

As shown for cyclic peptoids,^33^ the presence of two conformational enantiomers can be revealed by ^1^H NMR, using a chiral solvating agent (to form diastereomeric supramolecular complexes).^33^ Gradual addition of Pirkle’s reagent [(*R*)-1-(9-anthryl)-2,2,2-trifluoroethanol] to a racemic solution of **1a** and **1b** in deuterated chloroform induced the splitting of most proton resonances (Figure 2b,c). Variable-temperature NMR (VT NMR) experiments in C_2_D_2_Cl_4_ showed the stability of the conformers up to 373 K (Figure S9).

^1^H and ^13^C NMR analysis of cyclotrimer **2** and cyclic tetraamide **3** suggested, in analogy with the hexameric cyclic peptoids, closer in size, the contemporary presence of different conformers in slow equilibrium on the NMR time scale. Proof of their identity was obtained by HRMS and corroborated by VT NMR in DMSO and C_2_D_2_Cl_4_ solutions [600 MHz (Figures S10 and S11)] with simplification of the NMR spectra into a set of broad singlets (*T* = 373 K).

To our delight, 20-membered **3** gave single crystals suitable for X-ray diffraction analysis by slow evaporation from an acetonitrile solution. In the solid state, the macrocycle exhibits crystallographic inversion symmetry with the tertiary amide bonds in the *trans* conformation. Overall, it adopts a flat shape with the side chains extending horizontally with respect to the macrocyclic plane (Figure 3). Reciprocal carbonyl–carbonyl interactions^36,37^ emerge with short distances between the carbonyl groups of <3.22 Å (Figure S17) and O···C=O angles of 88.8(1)° and 81.5(1)° (Table S3), which are consistent with the values reported by Sarma and co-workers.^37^

**Figure 3:**
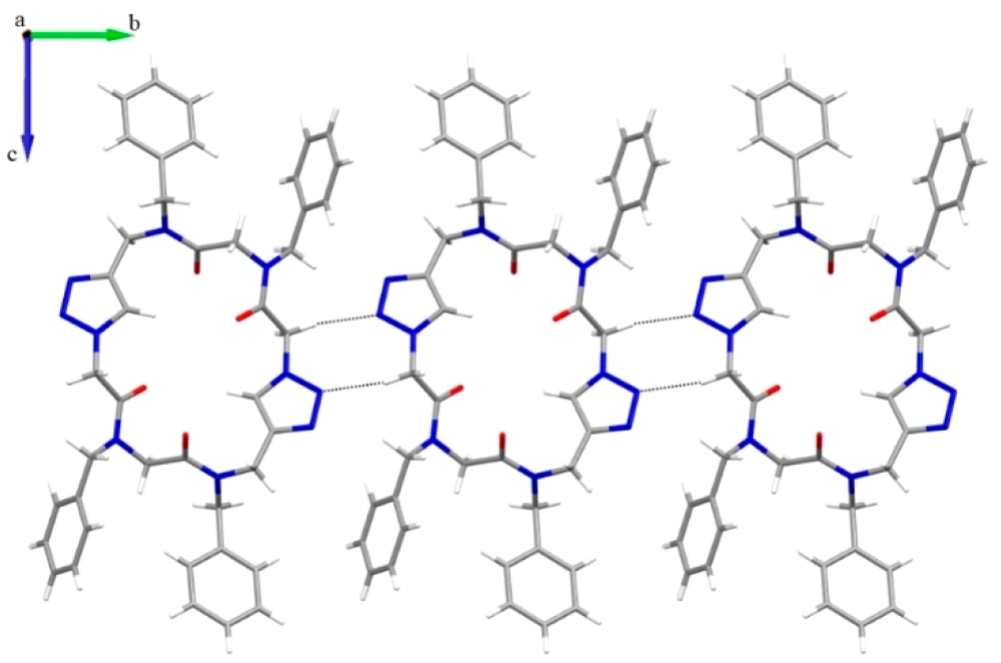
H-Bonded ribbon of **3** along the shortest *b* axis (as viewed along the *a* axis). H-Bonds are depicted as black dotted lines.

In the solid state, molecules of **3** align along the shortest *b* axis (Figure 3) through hydrogen bonds involving N4 of the triazole ring and the methylene hydrogen atom of the backbone [C5···N4, 3.407(3) Å; C5H5B···N4, 2.53 Å; C5H5B···N4, 150.4°]. This results in an amphiphilic layered structure, where the backbone atoms constitute the hydrophilic region and the benzyl side chains the hydrophobic region. Finally, the interlayer interactions are mainly characterized by hydrophobic interactions and H–H contacts involving the aromatic side chains. Notably, the peptidic analogue of compound **3** (CSD code OKECUC), reported by Ghadiri in 2003,^18d^ exhibited a completely different macrocycle conformation with the triazole moieties perpendicular to the plane of the macrocycle, featuring the tubular assembly of the macrocycles with ethanol molecules enclosed in the peptide nanotube.

In conclusion, we report the first members of a new class of intriguing “extended peptoid” macrocycles. Backbone conformations for smaller cyclodimer **1** and cyclic tetraamide **3** were supported by computational studies, NMR data, and X-ray diffraction analysis. The versatile synthetic approach of the linear precursors can afford precisely tailored macrocycles, both decorated with a wide range of side chains and containing 1,2,3-triazole regioisomeric rings. Combination of the complexation abilities of the cyclopeptoids with the coordination attitude of triazole moieties has provided access to a new class of potential host macrocycles. Studies of the synthesis of new derivatives and their complexation properties are in progress and will be reported in due course.

## Notes

The data underlying this study are available in the published article and its online Supporting Information.

## Supporting Information Available

Experimental procedures, characterization data, HPLC chromatograms of the new compounds, and DFT and crystallographic studies (PDF)

## Dedication

Dedicated to the memory of Piero Angela, a guide to the path of Science.
