# Supplementary material for: Macrocyclic Triazolopeptoids: A Promising Class of Extended Cyclic Peptoids
Source: Org Lett. 2022 Oct 12;24(42):7752–6. doi: 10.1021/acs.orglett.2c03062 (PMC9623583; doi:10.1021/acs.orglett.2c03062)
Supplement: Supplementary file 1 — ol2c03062_si_001.pdf [file ol2c03062_si_001.pdf]

Supporting Information

For

Macrocyclic triazolopeptoids: a promising class of extended cyclic peptoids

Alicja M. Araszczuk, Assunta D'Amato, Rosaria Schettini, Chiara Costabile, Giorgio Della Sala, Giovanni Pierri, Consiglia Tedesco, Francesco De Riccardis and Irene Izzo*

Department of Chemistry and Biology "A. Zambelli", University of Salerno, via Giovanni Paolo II, 132, Fisciano (SA), 84084, Italy

Corresponding author: Irene Izzo, iizzo@unisa.it

List of abbreviations

ACN: acetonitrile

Bn: benzyl

COSY: correlation spectroscopy

DCM: dichloromethane

DIC: *N*,*N*’-diisopropylcarbodimide

DIPEA: ethyldiisopropilamine

DMF: *N*,*N*’-dimethylformamide

DMSO: dimethyl sulfoxide

Fmoc: 9-fluorenylmethoxycarbonyl

HATU: *O*-(7-azabenzotriazol-1-yl)-*N*,*N*,*N'*,*N'*-tetramethyluronium hexafluorophosphate

HFIP: hexafluoroisopropanol

HMBC: heteronuclear multiple bond correlation

HMQC: heteronuclear multiple quantum coherence

HRMS: high resolution mass spectrometry

Ph: phenyl

RP HPLC: reversed-phase high-performance liquid chromatography

TCDE: tetrachlorodideuteroethane

TFA: trifluoroacetic acid

General procedures S4

1.0 Synthesis of macrocycles 1, 2 and 3 S5

1.1 Submonomer solid-phase synthesis of linear precursors **8**, **9** and **10** S5

1.1.1 Linear dimer **8** S6

1.1.2 Linear trimer **9** S7

1.1.3 Linear tetraoligamide **10** S8

1.2 General procedure for high dilution cyclization. Synthesis of macrocycles **1**, **2** and **3** S8

1.2.1 Cyclic dimer **1** S9

1.2.2 Cyclic trimer **2** S9

1.2.3 Cyclic tetraoligamide **3** S10

2.0 HPLC chromatograms ,^{1}H-, ^{13}C NMR and two-dimensional spectra of 1, 2 and 3 S11

2.1 HPLC chromatograms of **1**, **2** and **3** S12

2.2 1 D and 2 D spectra of compound **1**, **2** and **3** .S13

2.3 Procedure for the Pirkle's alcohol addition to racemic mixture **1a/1b** S20

2.4 ^{1}H NMR variable temperature experiment for **1**, **2** and **3** at high temperature and for **1** at low temperature. S22

2.5 ^{1}H NMR variable concentration experiments for **1** S25

3.0 Calculated structures and energies of dimer 1 S26

3.1 Computational details S28

3.2 Cartesian coordinates and energies S29

4.0 X-ray crystallography of cyclic tetraoligoamide 3 S34

4.1 Intramolecular CO···CO interactions S36

5.0 References and notes S37

General procedures

Starting materials and reagents, purchased from commercial suppliers, were used without purification unless otherwise mentioned. HPLC analyses were performed on a JASCO LC-NET II/ADC equipped with a JASCO Model PU-2089 Plus Pump and a JASCO MD-2010 Plus UV-vis multiple wavelength detector set at 220 nm. The column used was a C18 reversed-phase analytical column (Waters, Bondapak, 10 µm, 125 Å, 3.9 mm × 300 mm) run with linear gradients of ACN (0.1% TFA) into H_2O (0.1% TFA) over 30 min, at a flow rate of 1.0 mL/min for the analytical runs.

High resolution mass spectra (HRMS) were recorded on a Bruker Solarix XR (Bruker Daltonik GmbH, Bremen, Germany) Fourier transform ion cyclotron resonance mass spectrometer ((FTICR analyzer)) equipped with a 7T refrigerated actively shielded superconducting magnet, using matrix-assisted laser desorption/ionization (MALDI) Yields refer to chromatographically and spectroscopically (^{1}H- and ^{13}C NMR) pure materials.

NMR spectra were recorded on a Bruker DRX 600 (^{1}H at 600.13 MHz, ^{13}C at 150.90 MHz). Chemical shifts (δ) are reported in ppm relative to the residual solvent peak ($CHCl_3$, δ = 7.26; ^{13}CDCl3, δ = 77.00; C_2DHCl_4, TCDE, δ = 5.80; $(CH_3)_2SO$, DMSO, δ = 2.50; $(^{13}CH_3)_2SO$, DMSO, δ = 39.51) and the multiplicity of each signal is designated by the following abbreviations: s, singlet; d, doublet; t, triplet; m, multiplet; br, broad. 2D NMR experiments such as COSY, HSQC and HMBC were performed for the full assignment of each signal. Coupling constants (*J*) are quoted in Hertz. See list of abbreviations in the ESI.

1.0 Synthesis of macrocycles 1, 2 and 3

1.1 Submonomer solid-phase synthesis of linear precursors 8, 9 and 10

Solid-phase synthesis of liner precursors 8 and 9

2-chlorotrityl chloride resin (2,α-dichlorobenzhydryl-polystyrene cross-linked with 1% DVB; 100–200 mesh; 1.60 mmol g^{-1}, 0.100 g, 0.160 mmol) was washed with DCM (3 × 1 mL) and DMF (3 × 1 mL) and then swollen in dry DCM (1.0 mL) for 45 min. Azidoacetic acid, (handled carefully due to known explosive nature of azides), **4**, (0.026 g, 0.26 mmol) and DIPEA (139 µL, 0.80 mmol) in dry DCM (1.0 mL) were added to the resin and the vessel was stirred on a shaker platform for 60 min at room temperature. Then the resin was washed with DMF (3 × 1.0 mL), DCM (3 × 1.0 mL) and then with DMF (3 × 1.0 mL). Subsequent on-resin cycloaddition reaction was accomplished in the presence of copper iodide (0.046 g, 0.24 mmol), *N*-benzylprop-2-ynyl-1-amine, **5,** (0.046 g, 0.32 mmol), and DIPEA (1.4 mL, 8.00 mmol) in dry THF (1.5 mL). The resulting mixture was stirred overnight on the shaker platform at room temperature. Then the resin was washed with THF (5 x 1.0 mL), DMF (3 × 1.0 mL), DCM (3 × 1 mL) and with DMF (3 × 1.0 mL). Next, a solution of azidoacetic acid (0.162 g, 1.60 mmol) and DIC (273 µl, 1.76 mmol) in dry DMF (1.0 mL) was added to the resin and stirred on a shaker platform for 40 min at room temperature. Then the resin was washed again with DMF (3 × 1.0 mL), DCM (3 × 1.0 mL) and DMF (3 × 1.0 mL). After, the cycloaddition reaction was repeated following the procedure described above. The preparation of triazole-peptoid **2** needed an additional acylation and cycloaddition step. The synthesis proceeded until the target linear triazole-peptoids were obtained. The linear precursors were cleaved from the resin, previously washed with DCM (3 × 1.0 mL), by treatment with three aliquots of a solution of 20% HFIP in dry DCM (v/v; 3 × 1.0 mL), stirred on a shaker platform at room temperature for 30 min each time. The resin was filtered away and the combined filtrates were concentrated in vacuo. The final products were analyzed by MALDI mass spectrometry and RP-HPLC and used for the cyclization step without further purification.

Solid-phase synthesis of linear precursor 10

2-chlorotrityl chloride resin (0.100 g, 0.160 mmol) was washed with DCM (3 × 1.0 mL) and DMF (3 × 1.0 mL) and swollen in dry DCM (1 mL) for 45 min. Then the resin was acylated with azidoacetic acid, (**4**, 0.026 g, 0.26 mmol) in the presence of DIPEA (139 µL, 0.800 mmol) in dry DCM (1.0 mL). The mixture was stirred on a shaker platform for 60 min at room temperature. The resin was washed with DMF (3 × 1.0 mL), DCM (3 × 1.0 mL) and DMF (3 × 1.0 mL). The cycloaddition reaction was performed by adding copper iodide (0.046 g, 0.240 mmol), *N*-benzylprop-2-ynyl-1-amine (**5**, 0.046 g,

0.320 mmol), and DIPEA (1.4 mL, 8.00 mmol) in dry THF (1.5 mL) to the resin. The mixture was stirred overnight on the shaker platform at room temperature. Then the resin was washed with THF (5 x 1.0 mL), DMF (3 × 1.0 mL), DCM (3 × 1.0 mL) and DMF (3 × 1.0 mL). Subsequently, bromoacetic acid (**6**, 0.220 g, 1.60 mmol) and DIC (273 μL, 1.76 mmol) in dry DMF (1.0 mL) was added to the vessel and stirred on a shaker platform for 40 min at room temperature. Then the resin was washed again with DMF (3 × 1.0 mL), DCM (3 × 1.0 mL) and DMF (3 × 1.0 mL) and a solution of benzylamine, (**7**, 171 μL, 1.60 mmol) in dry DMF (1.0 mL) was added to the bromoacetylated resin and stirred on a shaker platform for 40 min at room temperature. The resin was washed with DMF (3 × 1.0 mL), DCM (3 × 1.0 mL) and DMF (3 × 1.0 mL). Afterwards, the oligomer sequence was expanded by acylation with azidoacetic acid, cycloaddition, bromoacetylation and substitution with benzylamine, following the protocol described above. The cleavage from the resin, previously washed with DCM (3 × 1.0 mL), was perfomed by treatment with three aliquots of a solution of 20% HFIP in dry DCM (v/v; 3 × 1.0 mL). The mixture was stirred on a shaker platform at room temperature for 30 min each time. The resin was filtered away and the combined filtrates were concentrated in vacuo. The final product was analyzed by MALDI mass spectrometry and RP-HPLC and used for the cyclization step without further purification.

1.1.1 Linear dimer 8

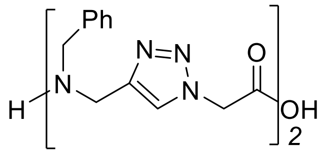

8: light yellow amorphous solid, 0.076 g, 100% yield;

HRMS (MALDI): *m/z* $[M + H]^+$ Calcd for $C_{24}H_{27}N_8O_3^+$ 475.2201; Found 475.2195;

HPLC: t_R: 5.8 min; 5 → 100% acetonitrile in 30 minutes (A: 0.1% TFA in water, B: 0.1% TFA in acetonitrile), flow: 1.0 ml/min, 220 nm.

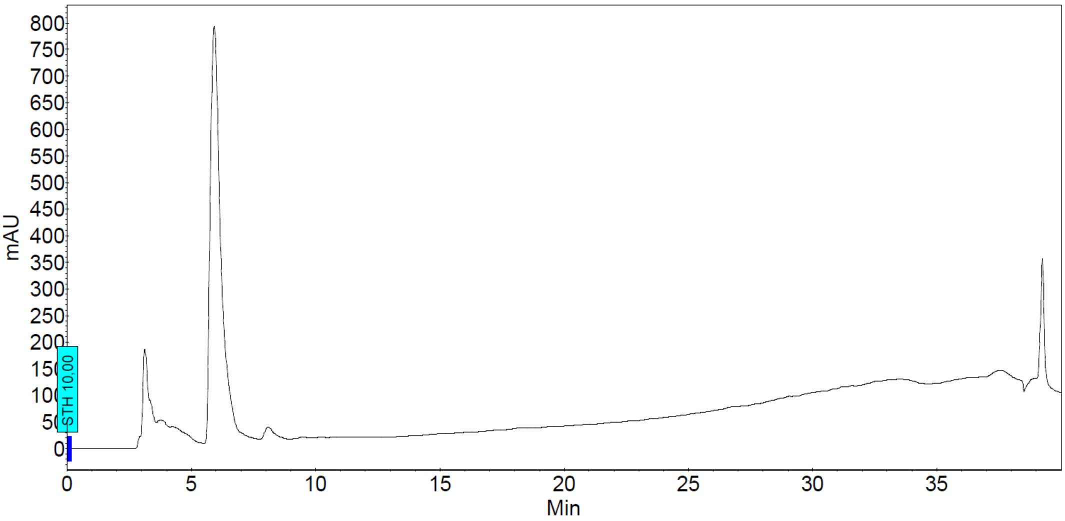

Figure S1 HPLC analysis of **8**

1.1.2 Linear trimer 9

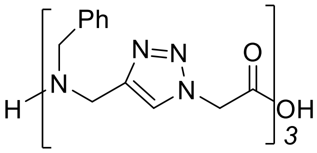

9: light yellow amorphous solid, 0.109 g, 100% yield;

HRMS (MALDI): *m/z* $[M + H]^+$ Calcd for $C_{36}H_{39}N_{12}O_4^+$ 703.3212; Found 703.3284;

HPLC: t_R: 7.6 min; conditions: 5 → 100% acetonitrile in 30 minutes (A: 0.1% TFA in water, B: 0.1% TFA in acetonitrile), flow: 1.0 ml/min, 220 nm.

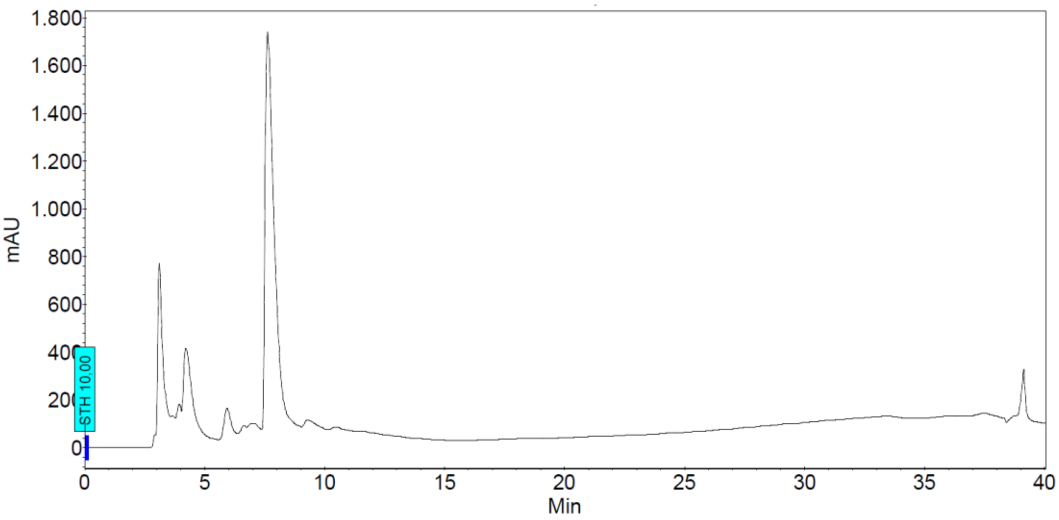

Figure S2 HPLC analysis of **9**

1.1.2 Linear tetramer 10

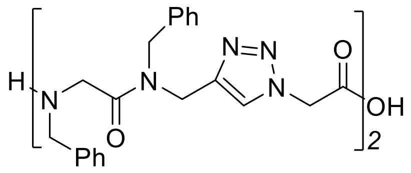

10: light yellow amorphous solid, 0.120 g, 100% yield;

HRMS (MALDI): *m/z* $[M + H]^+$ Calcd for $C_{42}H_{45}N_{10}O_5^+$ 769.3569; Found 769.3555;

HPLC: t_R: 8.2 min; conditions: 5 → 100% acetonitrile in 30 minutes (A: 0.1% TFA in water, B: 0.1% TFA in acetonitrile), flow: 1.0 mL/min, 220 nm.

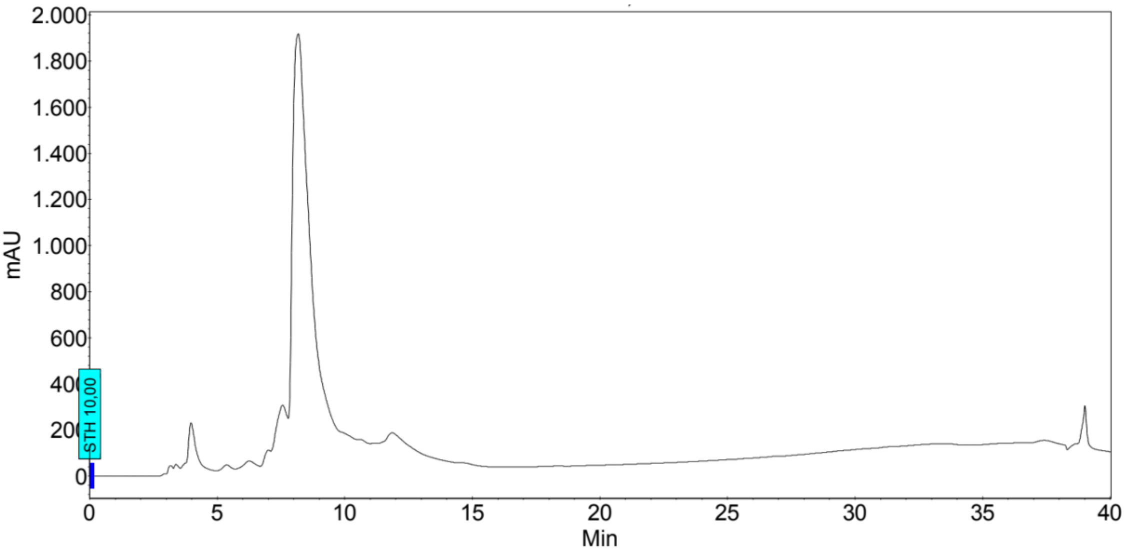

Figure S3 HPLC analysis of **10**

1.2. General procedure for high dilution cyclization. Synthesis of macrocycles 1, 2 and 3

To a stirred solution of HATU (0.243 g, 0.64 mmol) and DIPEA (170 μl, 1.00 mmol) in dry DMF (45 mL) at room temperature, a solution of a linear precursor (0.16 mmol) in dry DMF (8 mL) was added using a syringe pump in 3 h. After 16 h the resulting mixture was concentrated in vacuo, diluted with DCM (40 mL) and washed with 1 M HCl (2 × 20 mL). The aqueous layer was extracted with DCM (80 mL) and the combined organic phases were washed with water (60 mL), dried over $MgSO_4$ and concentrated in vacuo. The crude cyclic peptoids **1**, **2** and **3** were dissolved in hot acetonitrile and precipitated by slowly cooling the solution.

1.2.1 Compound 1

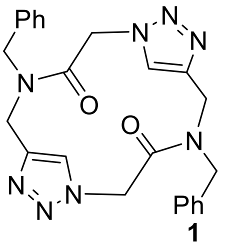

1

1: white amorphous solid, 0.025 g, 34% yield;

HRMS (MALDI): *m/z* $[M + H]^+$ Calcd for $C_{24}H_{25}N_8O_2^+$ 457.2095; Found 457.2105;

HPLC: t_R: 6.8 min.; conditions: 5 → 100% acetonitrile in 30 minutes (A: 0.1% TFA in water, B: 0.1% TFA in acetonitrile), flow: 1.0 mL/min, 220 nm;

^{1}H NMR (600 MHz, $CDCl_3$) δ: 7.84 (2H, s, C*H*NN), 7.45-7.32 (10H, m, Ar-*H*), 5.56 (2H, d, *J* 13.6 Hz, NNCH*H*CO), 5.29 (2H, d, *J* 14.3 Hz, BnNCH*H*C), 4.80 (2H, d, *J* 16.7 Hz, NCH*H*Ph), 4.75 (2H, d, *J* 13.6 Hz, NNC*H*HCO), 4.11 (2H, d, *J* 16.7 Hz, NC*H*HPh), 3.79 (2H, d, *J* 14.3 Hz, BnNC*H*HC);

^{13}C NMR (150 MHz, $CDCl_3$) δ: 166.0 × 2 (CH_2*C*O), 144.2 × 2 (CH_2*C*CHN), 135.7 × 2 (*C*-Ar), 129.2 × 4 (*C*-Ar), 128.2 × 2 (*C*-Ar), 127.0 × 4 (*C*-Ar), 122.4 × 2 (CH_2C*C*HN), 52.5 × 2 (NN*C*H_2CO), 50.9 × 2 (N*C*H_2Ph), 41.2 × 2 (BnN*C*H_2C).

1.2.2 Compound 2

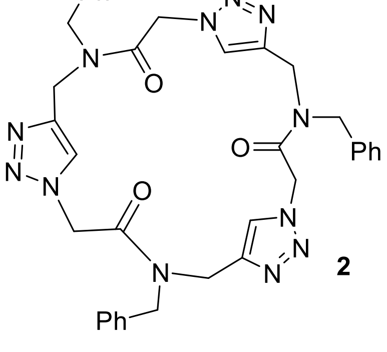

2

2: white amorphous solid, 0.031 g, 28% yield;

HRMS (MALDI): *m/z* $[M + H]^+$ Calcd for $C_{36}H_{37}N_{12}O_3^+$ 685.3106; Found 685.3094;

HPLC: t_R: 9.3 min.; conditions: 5 → 100% acetonitrile in 30 minutes (A: 0.1% TFA in water, B: 0.1% TFA in acetonitrile), flow: 1.0 mL/min, 220 nm;

^{1}H NMR (600 MHz, DMSO, mixture of rotamers) δ: 8.59-7.74 (3H, m), 7.42-7.29 (15H, m), 5.62-5.47 (6H, m), 4.78-4.47 (12H, m);

^{13}C NMR (150 MHz, DMSO, mixture of rotamers) δ: 166.4, 166.1, 144.0, 143.7, 143.5, 143.4, 143.1, 142.9, 136.8, 136.4, 128.8, 128.5, 127.8, 125.4, 125.0, 124.6, 51.0, 50.8, 50.6, 50.4, 50.3, 48.6, 48.4, 48.2, 42.6, 42.3, 41.9, 41.8, 41.7.

1.2.3 Compound 3

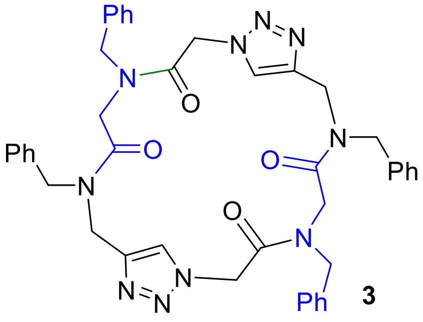

3

3: white amorphous solid, 0.029 g, 24% yield;

HRMS (MALDI): *m/z* $[M + H]^+$ Calcd for $C_{42}H_{43}N_{10}O_4^+$ 751.3463; Found 751.3491;

HPLC: t_R: 10.1 min; conditions: 5 → 100% acetonitrile in 30 minutes (A: 0.1% TFA in water, B: 0.1% TFA in acetonitrile), flow: 1.0 mL/min, 220 nm;

^{1}H NMR (600 MHz, $CDCl_3$, mixture of rotamers) δ: 8.66-8.02 (2H, m), 7.43-7.05 (20H, m), 5.40-5.33 (4H, m), 5.00-3.96 (16H, m);

^{13}C NMR (150 MHz, $CDCl_3$, mixture of rotamers) δ: 168.8, 168.7, 167.8, 166.8, 166.5, 165.7, 165.6, 146.8, 144.4, 143.8, 143.7, 143.2, 143.1, 136.5, 136.1, 135.0, 134.9, 129.3, 129.0, 128.8, 128.7, 128.6, 128.4, 128.2, 127.9, 127.8, 127.7, 127.1, 126.9, 126.8, 126.5, 126.4, 126.1, 126.0, 125.7, 125.5, 52.6, 52.4, 52.2, 51.8, 51.3, 51.1, 51.0, 50.6, 50.5, 50.2, 50.0, 49.8, 48.6, 48.4, 48.3, 47.7, 47.5, 47.3, 47.2, 43.5, 43.3, 43.1, 42.9, 42.6, 42.3.

2.0 HPLC chromatograms ,^{1}H-, ^{13}C NMR and two-dimensional spectra of 1, 2 and 3

2.1 HPLC chromatograms of 1, 2 and 3

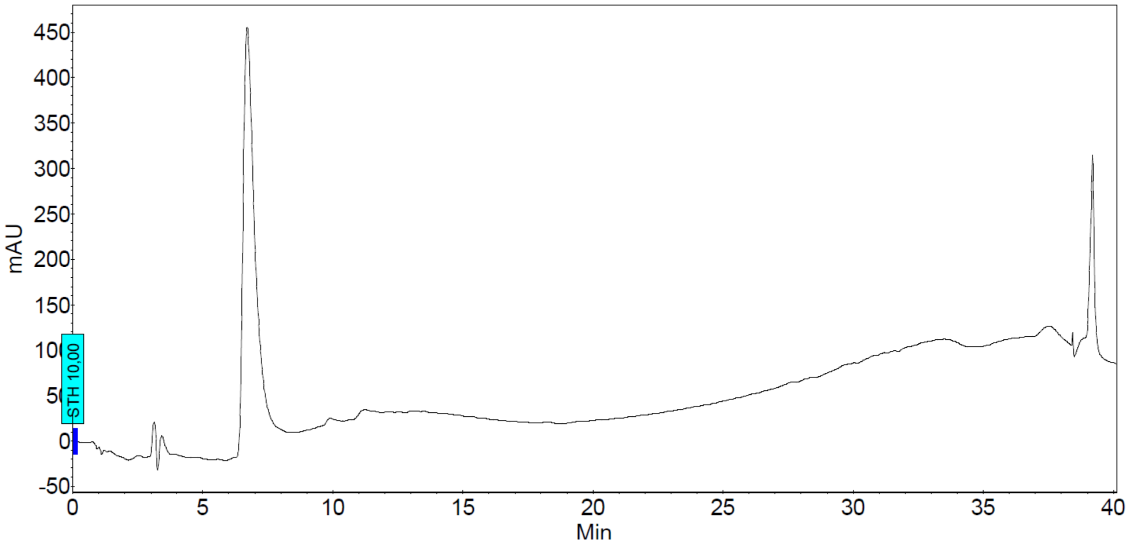

Figure S4 HPLC analysis of **1**

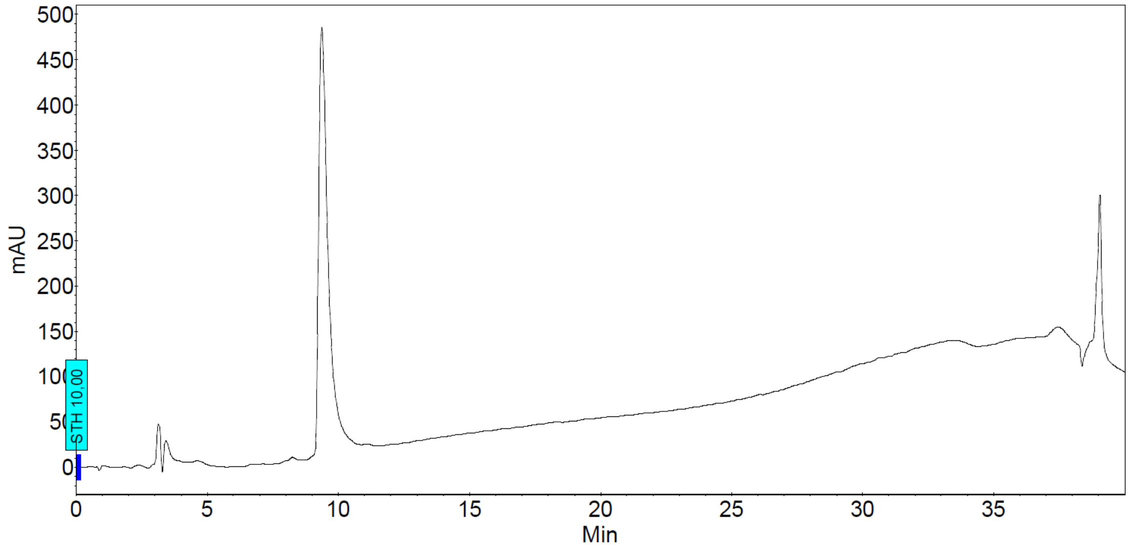

Figure S5 HPLC analysis of **2**

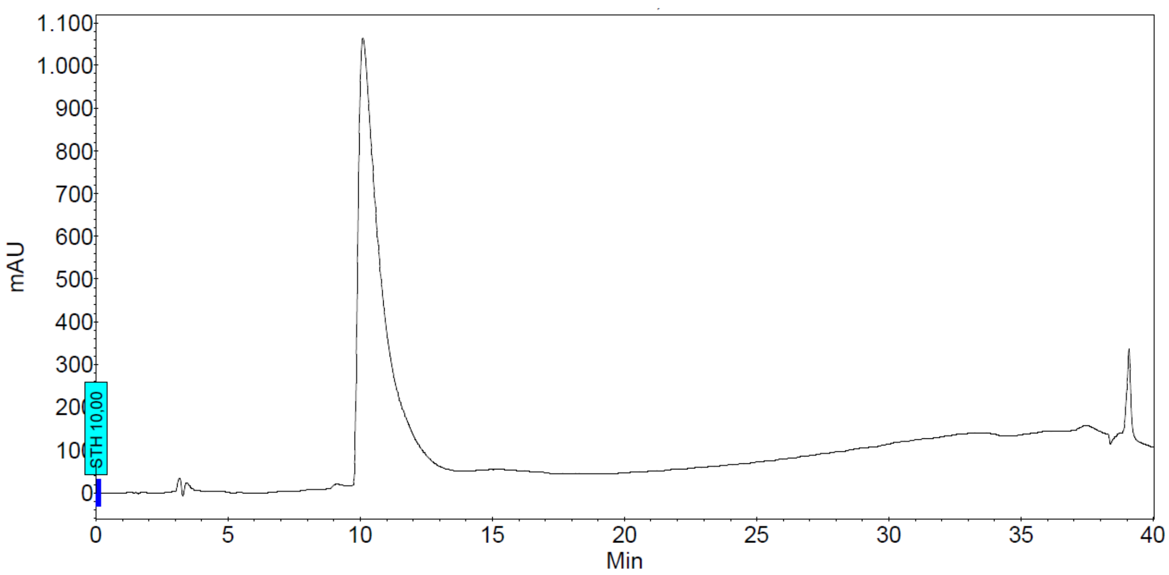

Figure S6 HPLC analysis of **3**

2.2 1 D and 2 D spectra of compound 1, 2 and 3

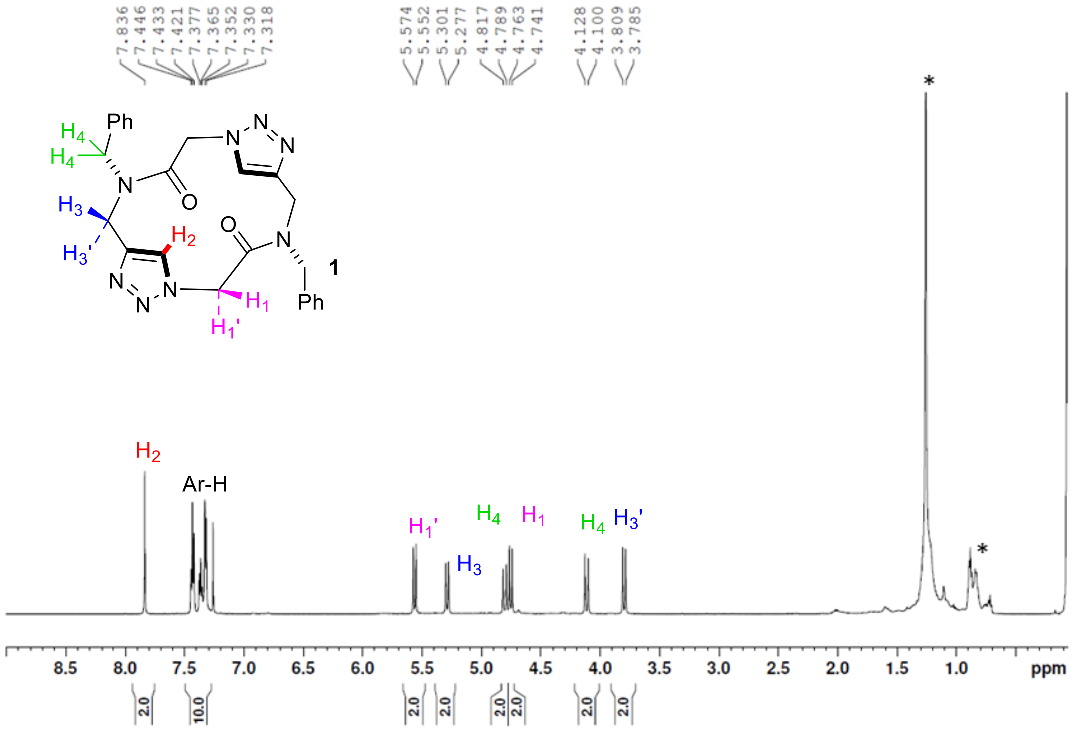

1:^{1}H NMR (600 MHz, $CDCl_3$); Water and grease impurities are labelled with asterisks.

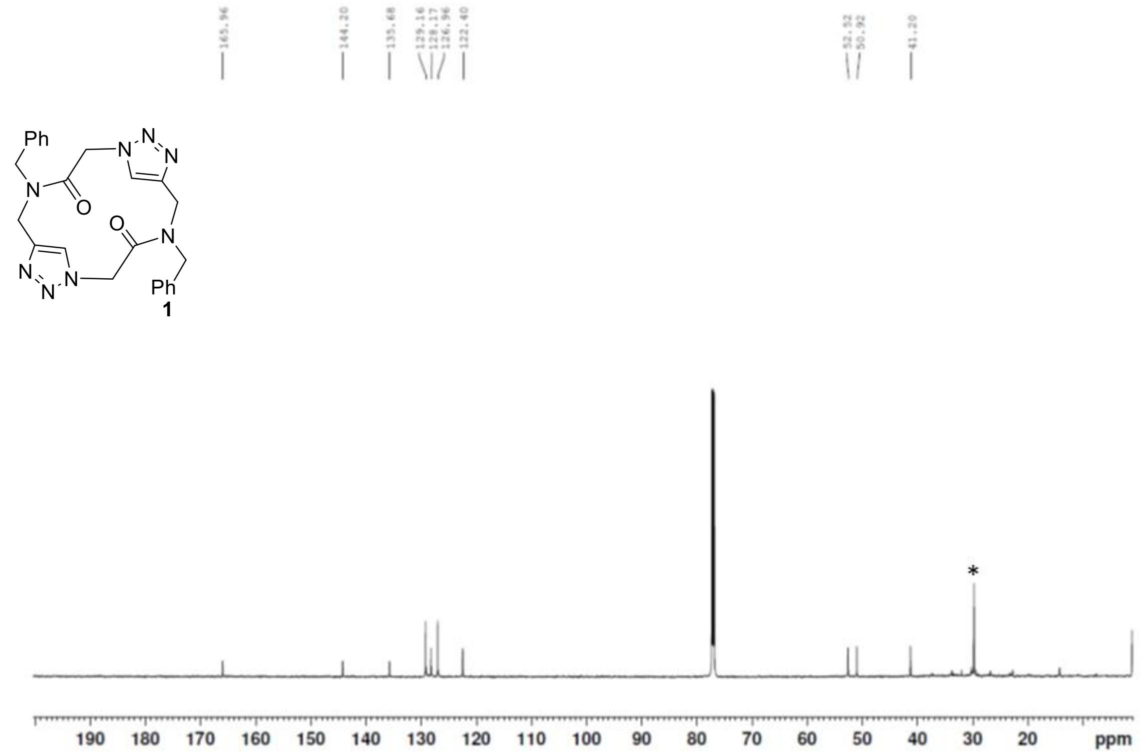

1:^{13}C NMR (150 MHz, $CDCl_3$); Grease impurity is labelled with an asterisk.

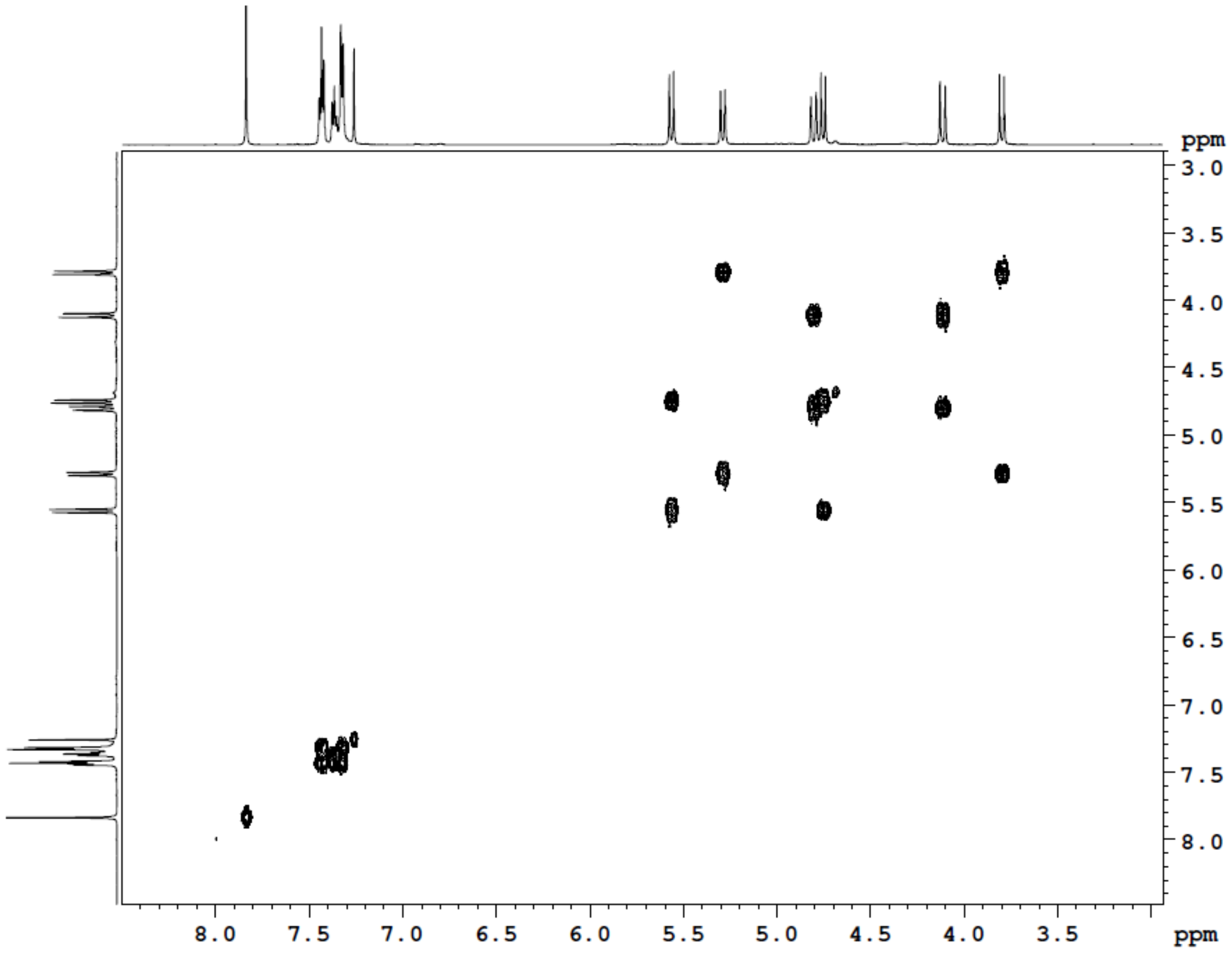

1: COSY NMR (600 MHz, $CDCl_3$)

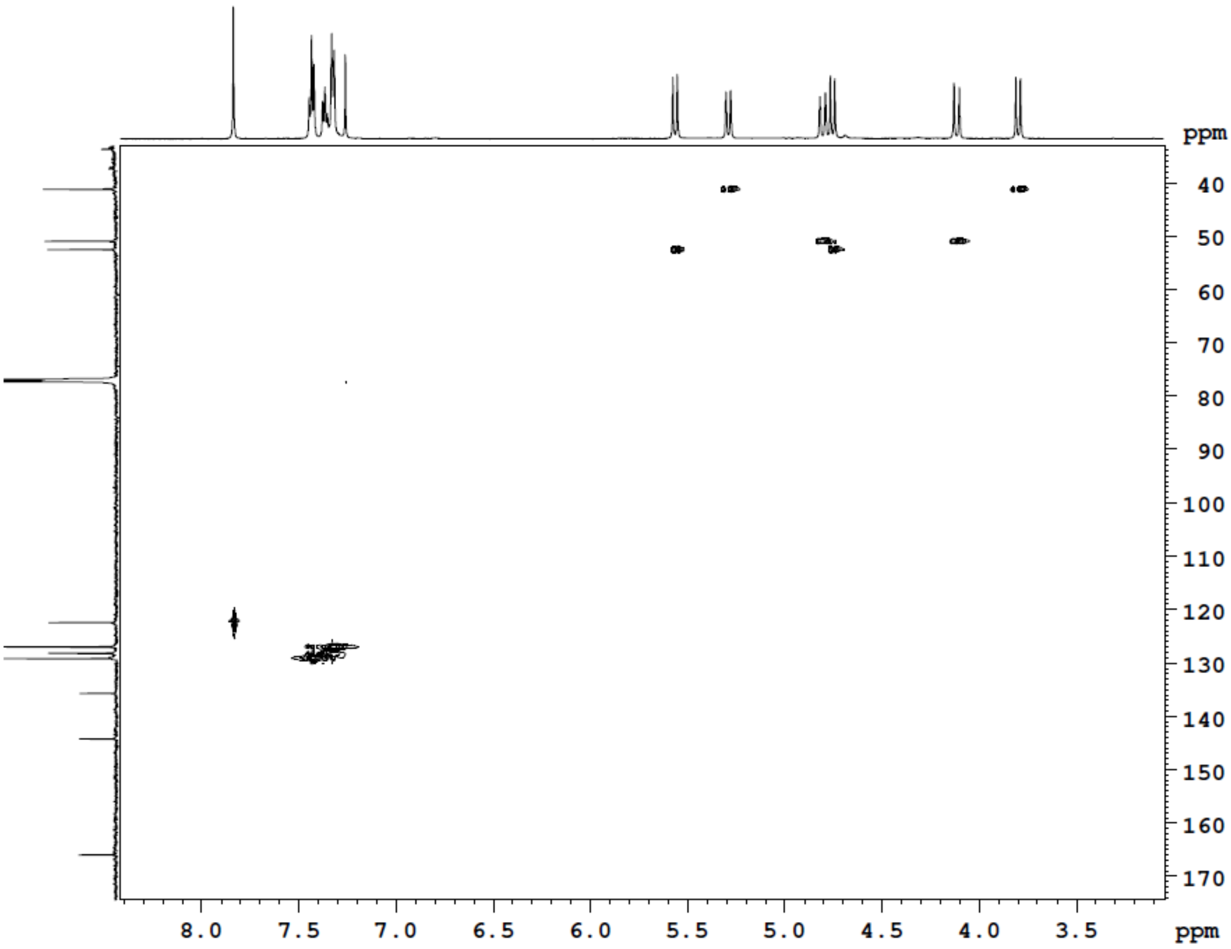

1: HSQC NMR (600 MHz, $CDCl_3$)

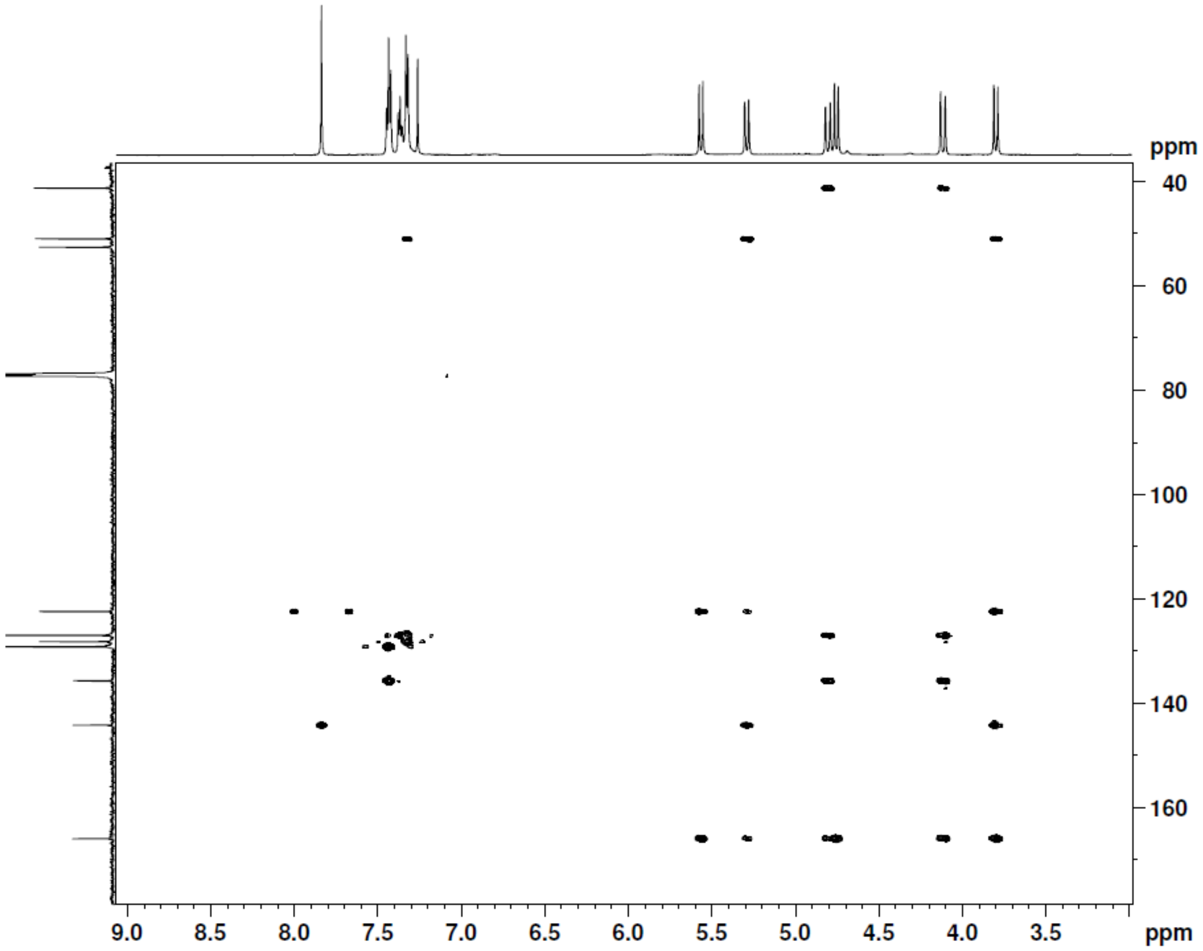

1: HMBC NMR (600 MHz, $CDCl_3$)

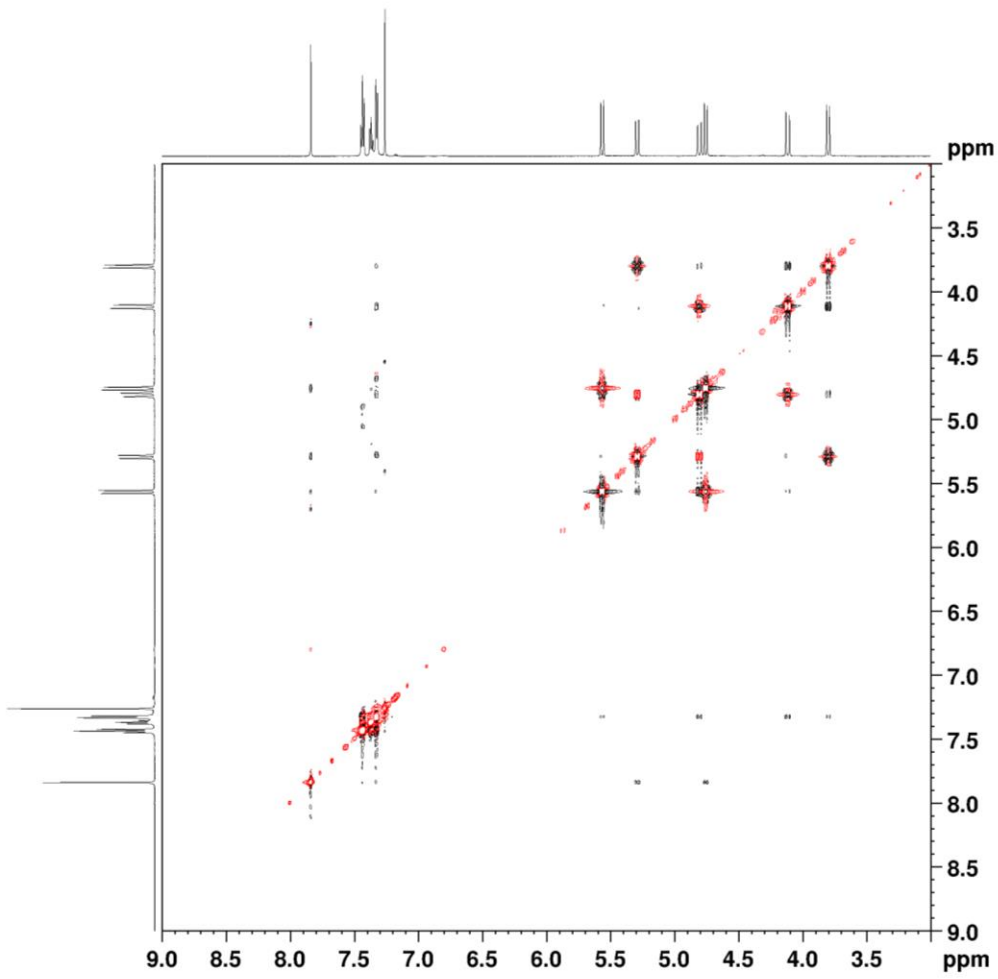

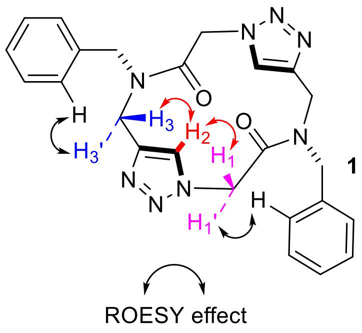

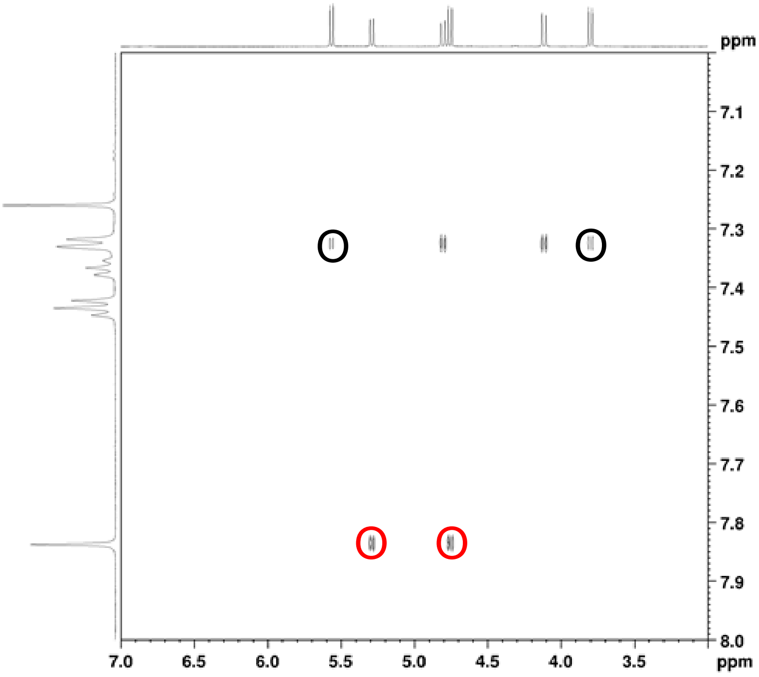

1: ROESY NMR (600 MHz, $CDCl_3$)

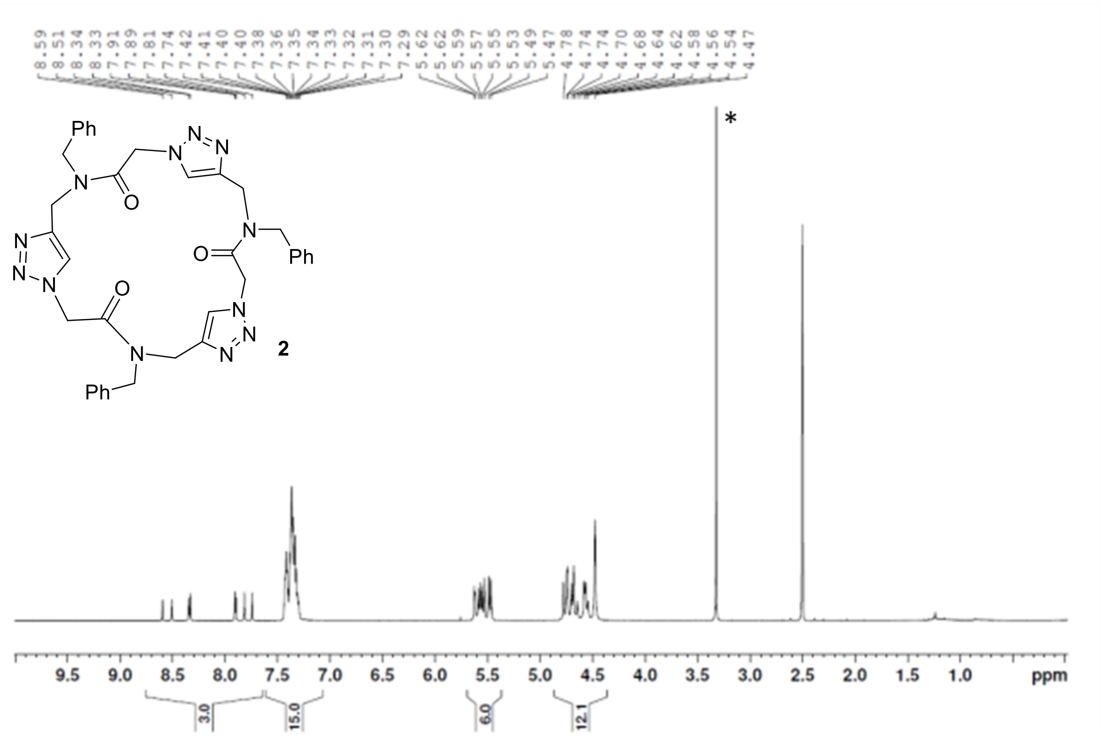

2:^{1}H NMR (600 MHz, DMSO); Water impurity is labelled with an asterisk.

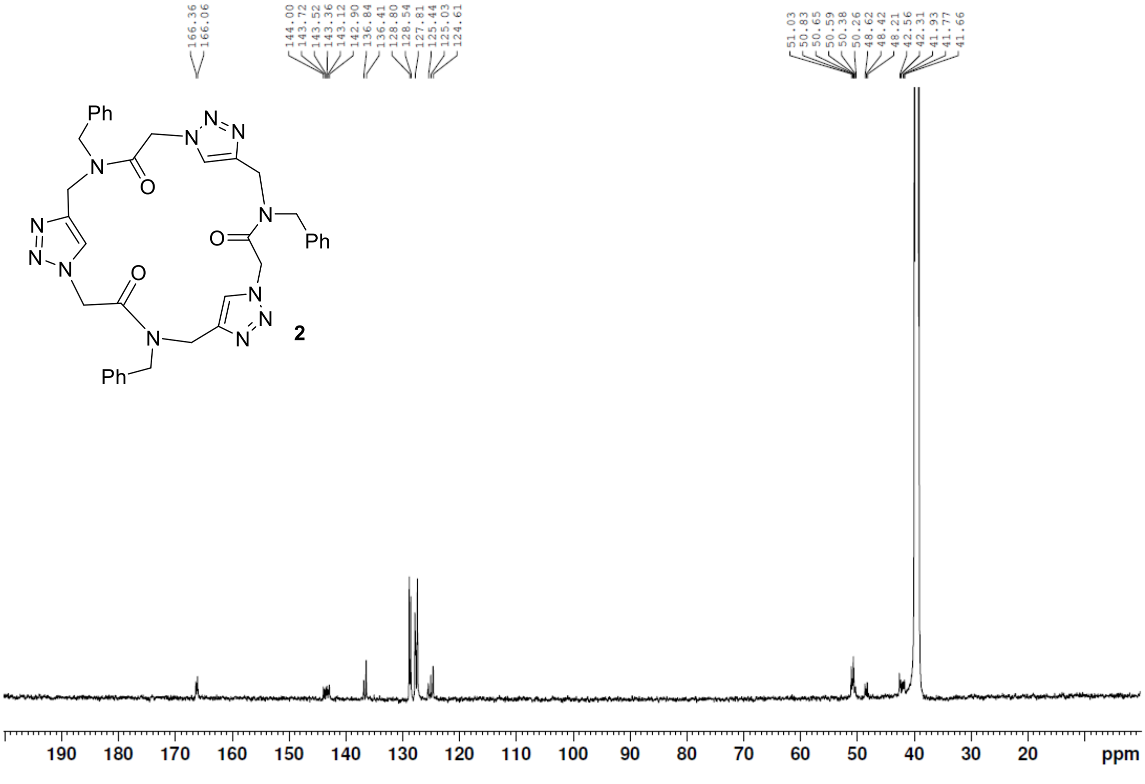

2:^{13}C NMR (150 MHz, DMSO)

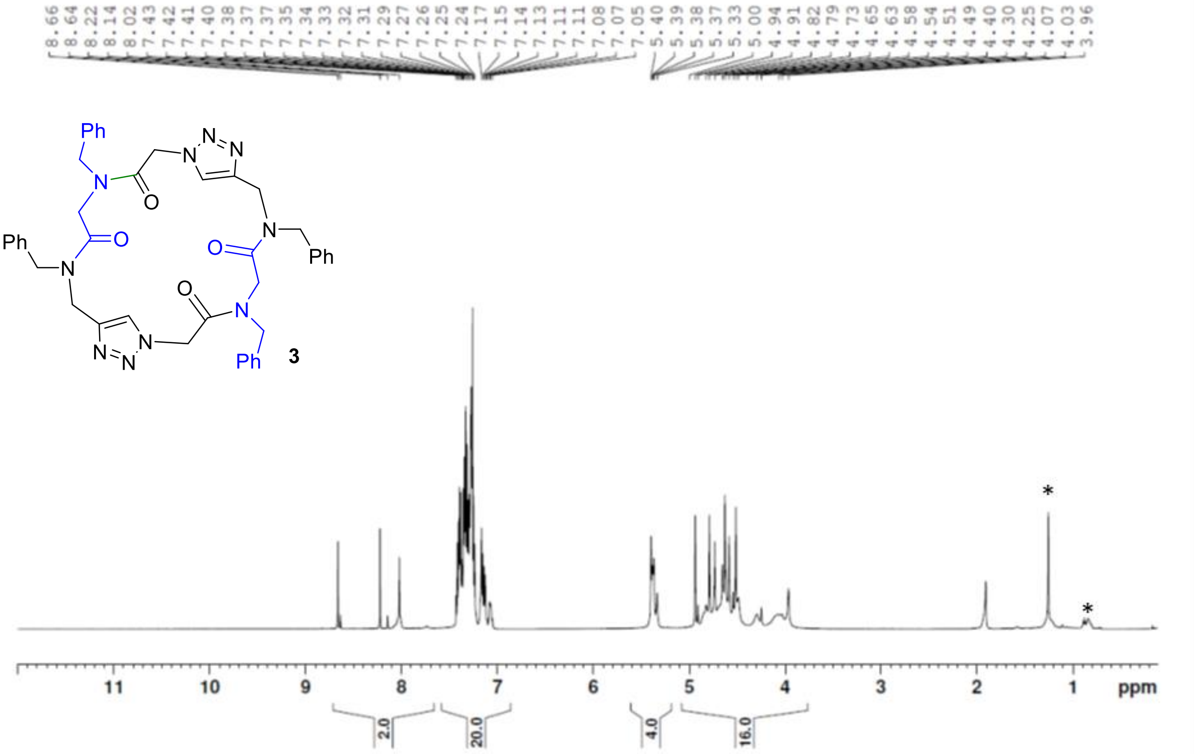

3:^{1}H NMR (600 MHz, $CDCl_3$); Water and grease impurities are labelled with asterisks.

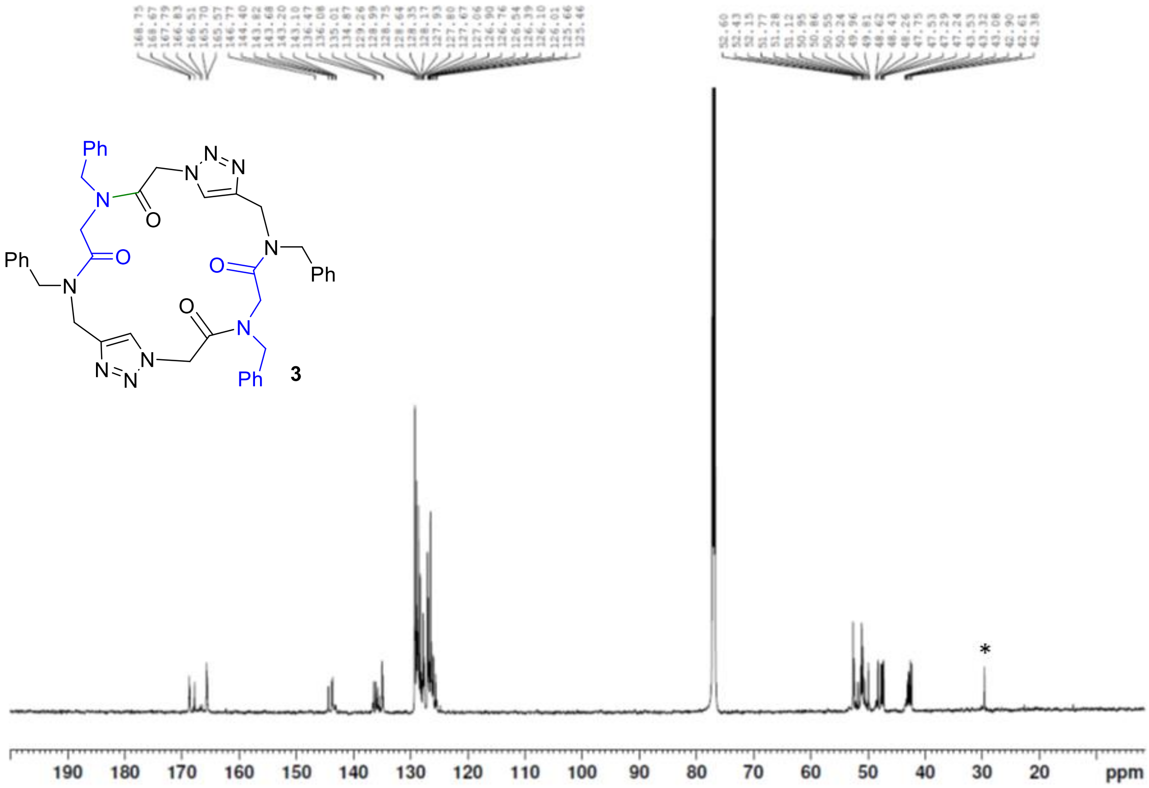

3:^{13}C NMR (150 MHz, $CDCl_3$); Grease impurity is labelled with an asterisk.

2.3 Procedure for the Pirkle's alcohol addition to racemic mixture 1a/1b

To a 8.0 mM solution of cyclic triazolopeptoid **1a/1b** in $CDCl_3$ (0.5 mL), 0.5 equivalents of Pirkle's alcohol ((*R*)-1-(9-anthryl)-2,2,2-trifluoroethanol) were added. After the addition the mixture was mixed for 1 minute and the ^{1}H NMR spectrum was recorded. Further 1, 2, and 3 equivalents of Pirkle's alcohol were added in order to increase the protons resonances' splitting (as reported in Figure S7).

NMR spectra were recorded on a Bruker DRX 600 (^{1}H at 600.13 MHz). The residual solvent peak was $CHCl_3$, set at δ = 7.26.

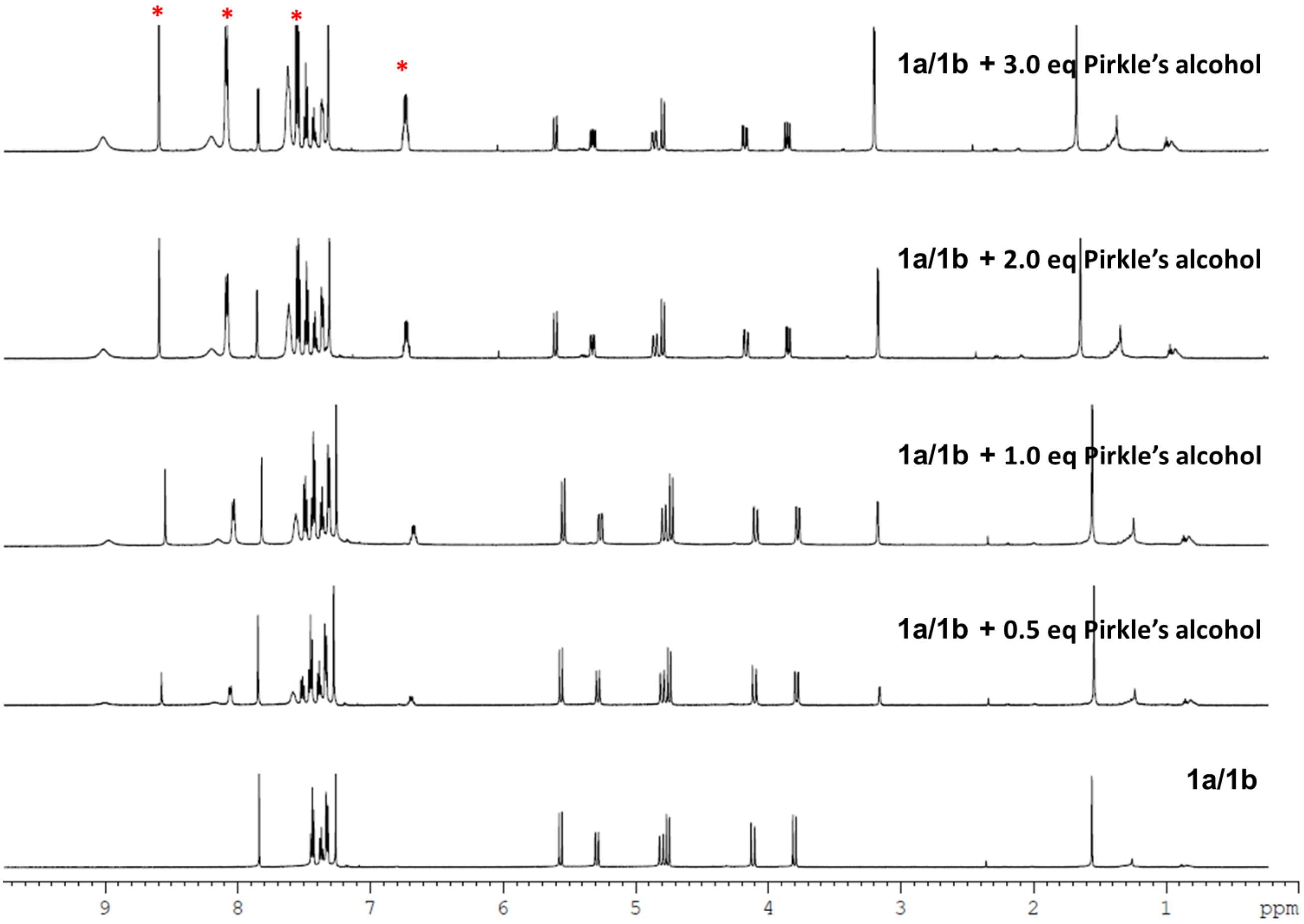

Figure S7. Quantitative step-wise addition of Pirkle's alcohol to 1a/1b. ^{1}H NMR (600 MHz, $CDCl_3$, 298 K, 8.0 mM solution). Full spectra. *Indicates the Pirkle's alcohol resonances.

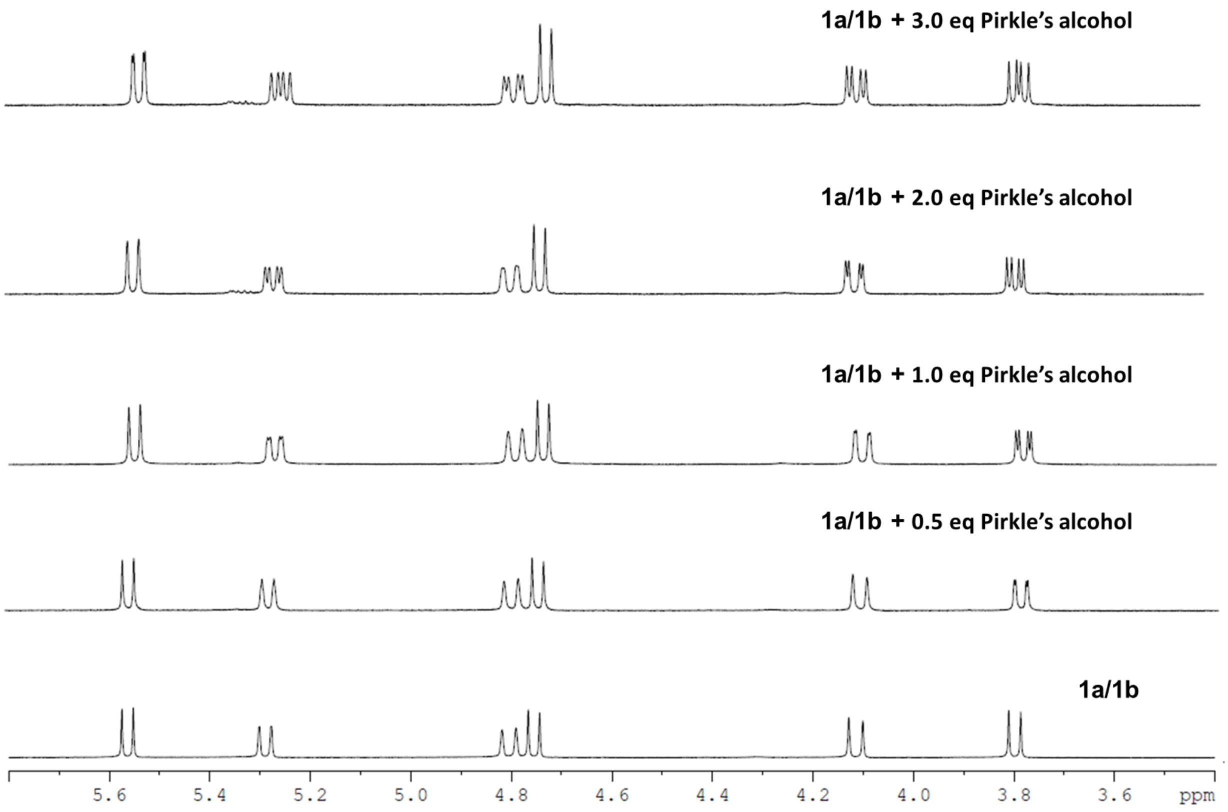

Figure S8. Quantitative step-wise addition of Pirkle's alcohol to 1a/1b. ^{1}H NMR (600 MHz, $CDCl_3$, 298 K, 8.0 mM solution). Expansion of ^{1}H NMR 5.8-3.4 ppm.

2.4 ^{1}H NMR variable temperature experiment for 1, 2 and 3 at high temperature and for 1 at low temperature.

2.4.1 High temperature experiments

Cyclic triazolopeptoids **1** and **3** were dissolved in $C_2D_2Cl_4$ (TCDE, 5.0 mM solution) and **2** was dissolved in DMSO (5.0 mM solution). Then ^{1}H NMR spectra were acquired at different temperatures, increasing 10 or 20 K each time.

For the compounds **1**, **2** and **3** no coalescence was observed up to 373 K.

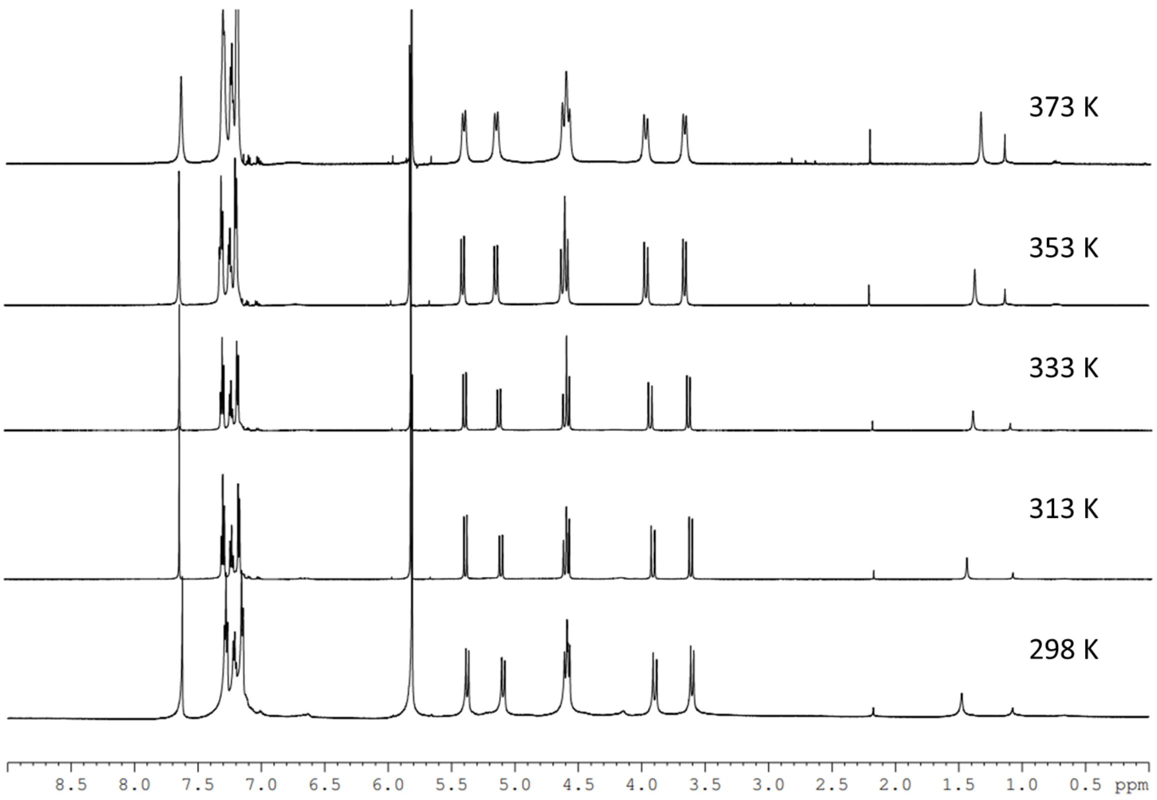

Figure S9. Variable temperature ^{1}H NMR spectra of compound **1** (600 MHz, $C_2D_2Cl_4$, 5.0 mM solution).

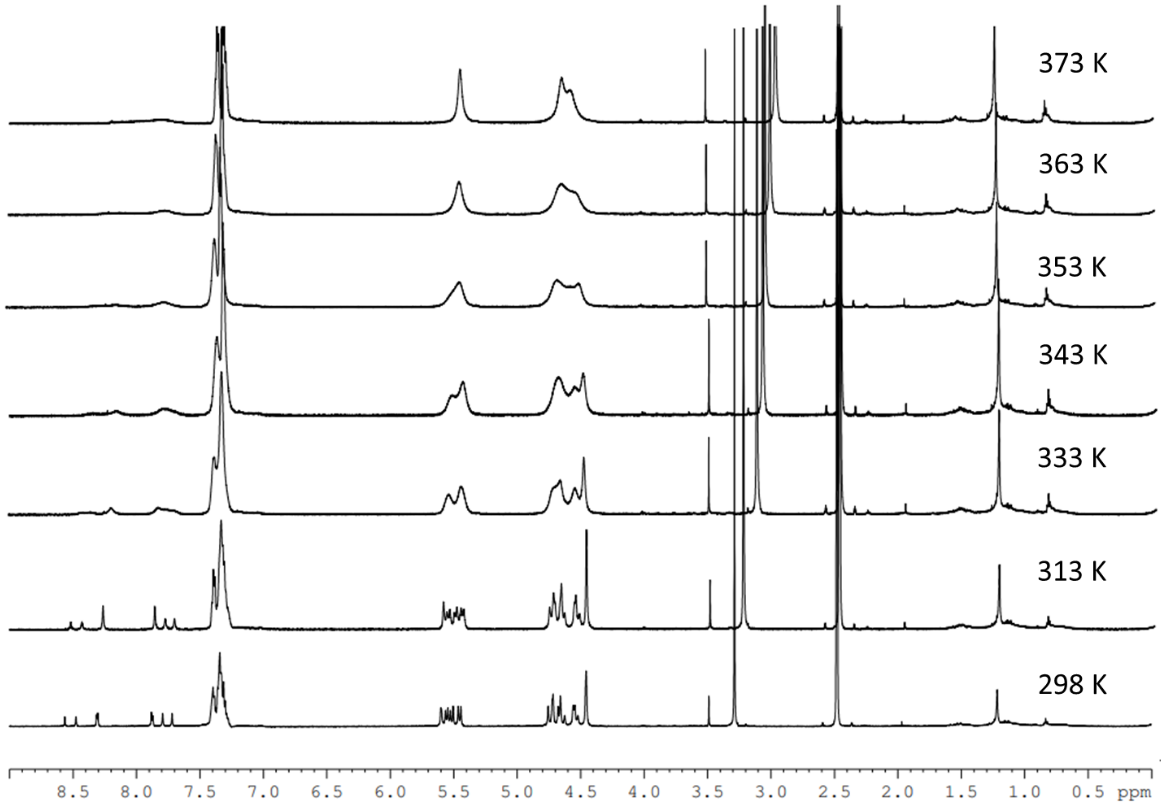

Figure S10 Variable temperature ^{1}H NMR spectra of compound **2** (600 MHz, DMSO, 5.0 mM solution).

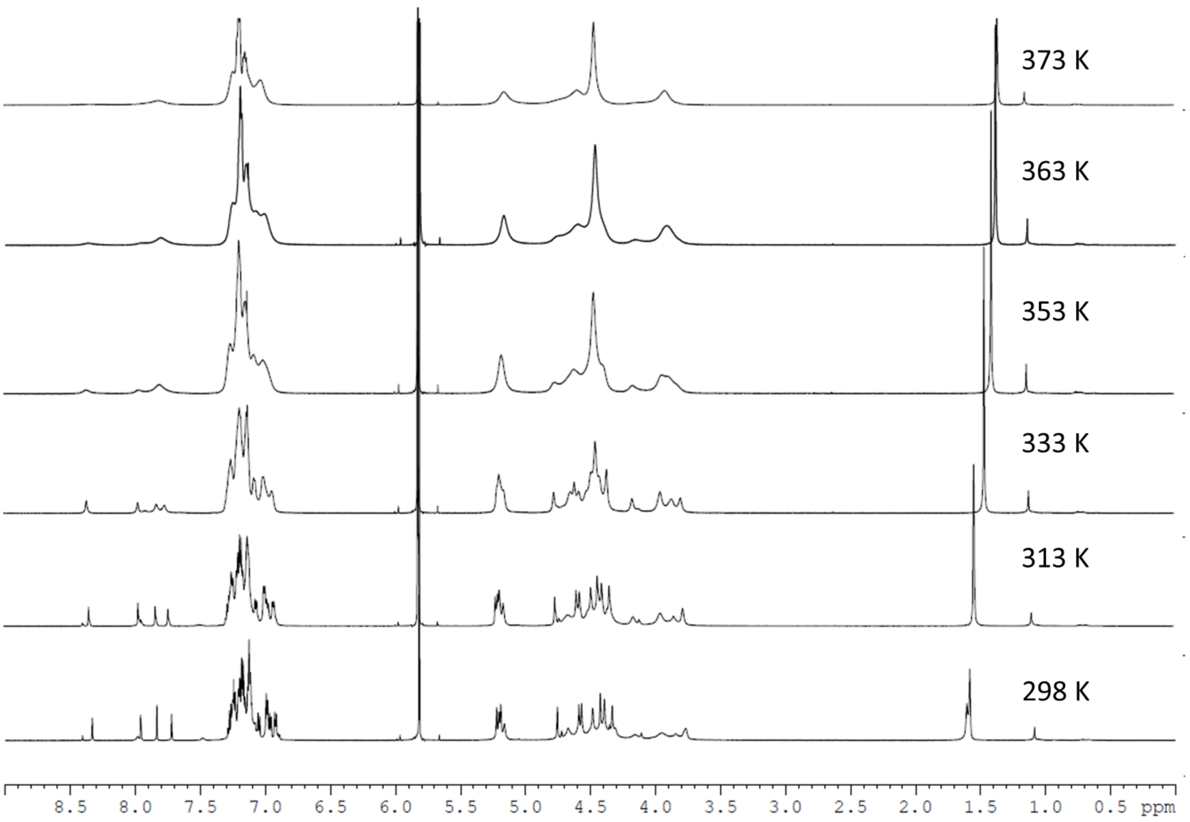

Figure S11. Variable temperature ^{1}H NMR spectra of compound **3** (600 MHz, $C_2D_2Cl_4$, 5.0 mM solution).

2.4.2 Low temperature experiment

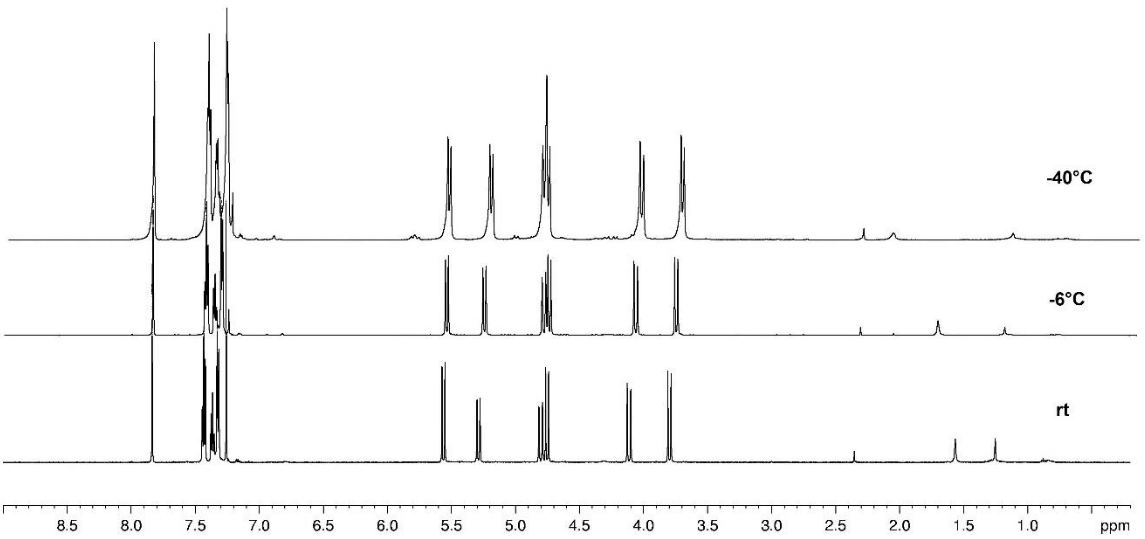

Figure S12. Variable temperature ^{1}H NMR spectra of compound **1** (600 MHz, $CDCl_3$, 16.0 mM solution).

2.5 ^{1}H NMR variable concentration experiments for 1

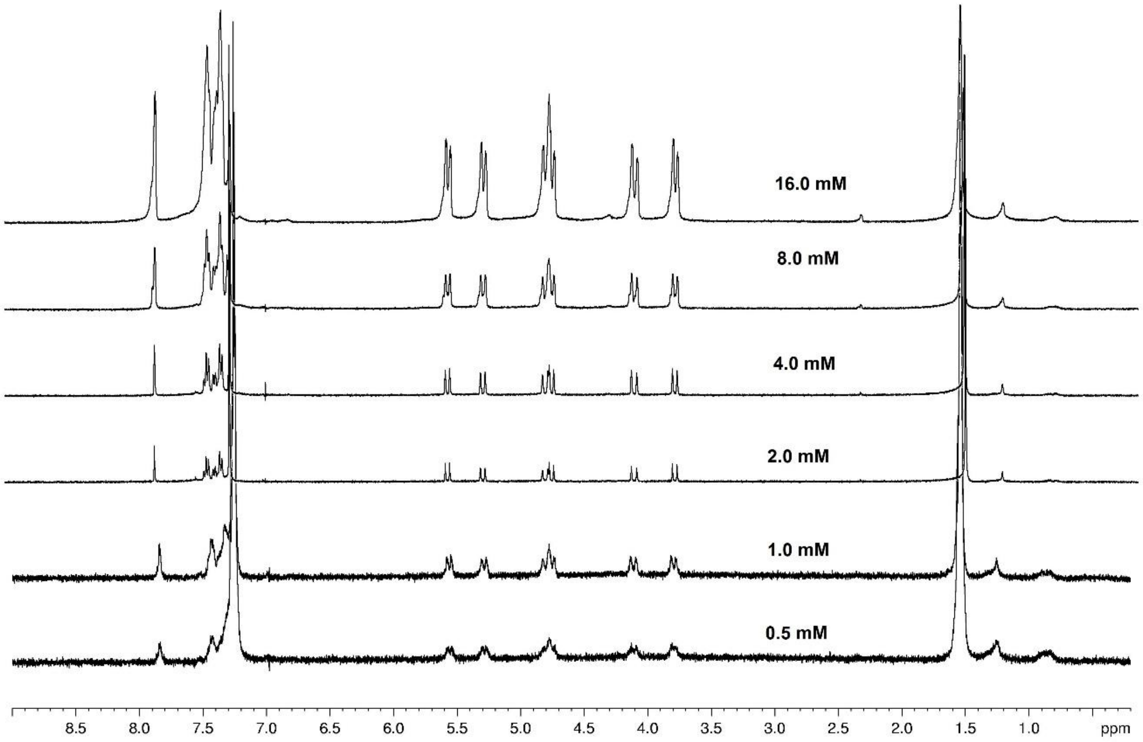

Figure S13. ^{1}H NMR spectra: variable concentrations of compound **1** (400 MHz, $CDCl_3$).

3.0 Calculated structures and energies of cyclodimer 1

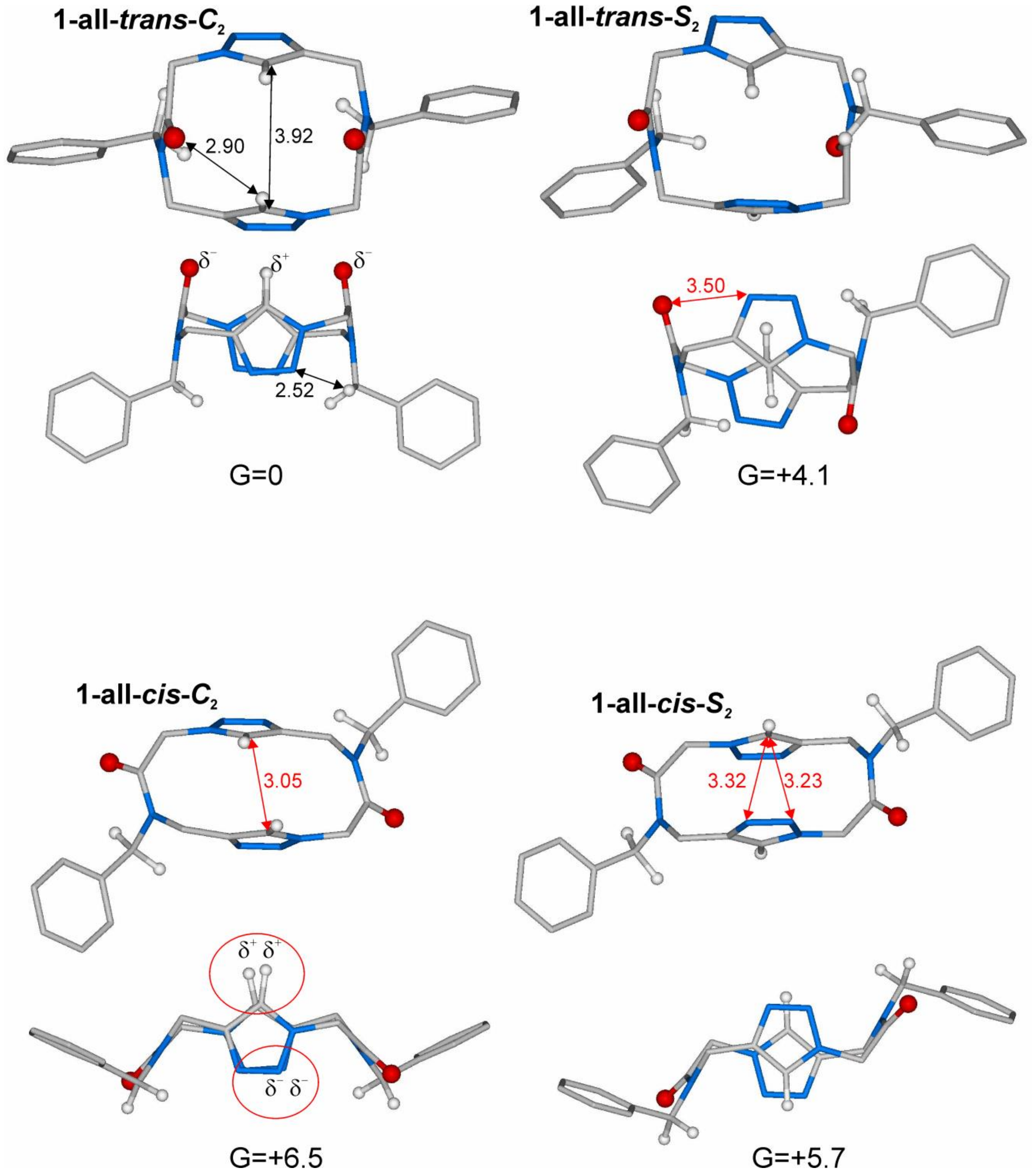

Figure S14. Top and side view of minimum energies structures of all *trans* S_2 or C_2 conformations and all *cis* S_2 or C_2 conformations of dimer **1**. Free energies, calculated in $CHCl_3$ at the BP84/TZVP level, are in kcal/mol. Distances are in Å (in red distances of repulsive interactions and in black distances of attractive or non-repulsive interactions). Non-relevant hydrogens were omitted for clarity.

Table S1. Internal (E) and free energies (G) of minimum energy structures of Figure S14 in gas fase and $CHCl_3$.

	E(gas)	G(gas)	E($CHCl_3$)	G($CHCl_3$)
**1-all-*trans*-*C*$_2$**	0	0	0	0
**1-all-*trans*-*S*$_2$**	7.4	6.2	5.2	4.1
**1-all-*cis*-*C*$_2$**	13.1	11.1	8.5	6.6
**1-all-*cis*- *S*$_2$**	10.8	8.7	7.8	5.7

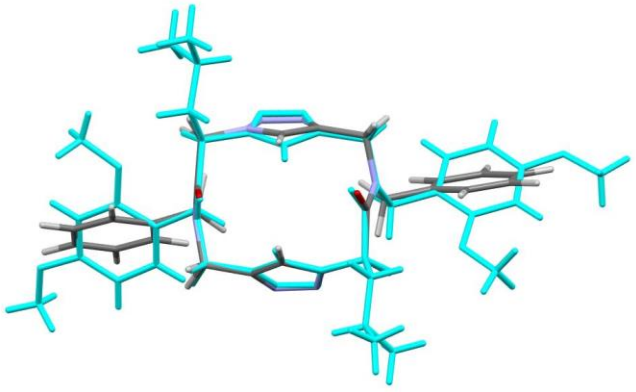

Figure S15. Backbone atoms overlay between the most stable conformer of **1** and the analogous cyclic dimer pseudo-tetrapeptide by Ghadiri and coworkers (cyan, CSD code SURWUY, compound 4 in ref. 18b). RMSD is 0.163 Å.

3.1 Computational details

The DFT calculations were performed with the Gaussian09 set of programs,[3] using the BP86 functional of Becke and Perdew.[4] The electronic configuration of the molecular systems was described with the standard triple zeta valence basis set with a polarization function of Ahlrichs and co-workers for H, C, N and O (TZVP keyword in Gaussian).[5] The geometry optimizations were performed without symmetry constraints, and the characterization of the located stationary points was performed by analytical frequency calculations. Solvent effects including contributions of non-electrostatic terms have been estimated in single-point calculations on the gas phase optimized structures, based on the polarizable continuous solvation model PCM using $CHCl_3$ as a solvent.[6]

3.2 Cartesian Coordinates and energies of calculated structures

58

1-all-*trans*-*C*$_2$ E(gas)=-1517.91956665 G(gas)=-1517.531273 E($CHCl_3$)=-1517.93490348

C	1.280746	-0.878728	2.074820
C	0.243631	-1.789569	1.949174
C	2.708568	-1.005827	1.633182
N	-0.866863	-1.089343	2.309366
H	0.208799	-2.821430	1.616982
N	-0.542470	0.198348	2.642379
C	-2.268837	-1.481129	2.161813
N	0.756756	0.316995	2.503141
C	-2.535175	-1.778921	0.667671
H	-2.455639	-2.412541	2.710908
H	-2.871266	-0.674287	2.595859
O	-2.410045	-2.943428	0.268536
N	-2.798024	-0.733314	-0.174862
C	-2.708808	-1.007320	-1.632643
C	-2.896419	0.684070	0.198082
C	-1.281040	-0.880244	-2.074523
H	-3.097654	-2.015715	-1.815864
H	-3.337774	-0.273081	-2.153534
C	-0.243928	-1.791052	-1.948527
N	-0.757049	0.315300	-2.503304
N	0.866577	-1.090985	-2.308971
H	-0.209104	-2.822760	-1.615849
N	0.542190	0.196616	-2.642460
C	2.268506	-1.482705	-2.160944
C	2.534690	-1.779234	-0.666516
H	2.455285	-2.414620	-2.709196
H	2.871057	-0.676319	-2.595671
O	2.409042	-2.943333	-0.266359
N	2.797979	-0.733041	0.175148
C	2.896810	0.684026	-0.198952
H	3.337126	-0.270811	2.153465
H	3.097863	-2.013863	1.817438
C	-4.295945	1.261582	0.040171
H	-2.538894	0.820664	1.228315
H	-2.191829	1.247710	-0.436193
C	-4.447346	2.613654	-0.306240
C	-5.718783	3.187655	-0.400737
C	-6.858827	2.413092	-0.158689
C	-6.717728	1.062149	0.176066
C	-5.444348	0.490809	0.274710
H	-3.560131	3.220454	-0.507693
H	-5.819467	4.240486	-0.673724
H	-7.852737	2.858750	-0.237485
H	-7.602560	0.448186	0.359052
H	-5.343133	-0.568561	0.522918
C	4.296388	1.261363	-0.041031
H	2.539749	0.819794	-1.229443
H	2.192095	1.248359	0.434558

C	4.447996	2.613452	0.305197
C	5.719527	3.187269	0.399622
C	6.859447	2.412500	0.157679
C	6.718142	1.061522	-0.176876
C	5.444683	0.490369	-0.275437
H	3.560876	3.220418	0.506569
H	5.820378	4.240117	0.672480
H	7.853423	2.858026	0.236400
H	7.602882	0.447399	-0.359771
H	5.343297	-0.569035	-0.523433

58

1-all-*trans*-*S*$_2$ E(gas)=-1517.90783782 G(gas)=-1517.521357 E($CHCl_3$)=-1517.92658637

C	0.706033	2.144980	-1.050725
C	0.111182	2.030766	0.195957
C	2.013748	1.592237	-1.558318
N	-1.086358	2.667489	0.058172
H	0.407517	1.552322	1.124301
N	-1.228464	3.159343	-1.208715
C	-2.169396	2.802011	1.032267
N	-0.141497	2.842552	-1.871855
C	-2.394003	1.497470	1.834525
H	-1.919764	3.569779	1.776303
H	-3.052951	3.133903	0.468928
O	-2.225911	1.513966	3.053296
N	-2.711524	0.351365	1.135963
C	-2.457070	-0.948949	1.806217
C	-3.020762	0.309551	-0.297997
C	-1.043491	-1.392368	1.555584
H	-2.606267	-0.805313	2.883234
H	-3.191818	-1.673915	1.430157
C	-0.458281	-1.880880	0.397118
N	-0.045013	-1.161543	2.473195
N	0.876185	-1.908551	0.668681
H	-0.851177	-2.181265	-0.567992
N	1.114205	-1.461313	1.941682
C	1.980961	-2.021981	-0.285490
C	1.868345	-0.814129	-1.246385
H	2.909694	-2.070451	0.295376
H	1.869242	-2.935213	-0.882602
O	1.195820	-0.925243	-2.274195
N	2.418134	0.374560	-0.834389
C	3.304700	0.503606	0.335054
H	2.826470	2.328967	-1.464150
H	1.892715	1.353450	-2.623704
C	4.725018	-0.004967	0.123178
H	3.337559	1.576661	0.582476
H	2.855690	0.005755	1.210010
C	5.493065	-0.361863	1.244009
C	6.816715	-0.786482	1.094281
C	7.388970	-0.865282	-0.180582
C	6.627755	-0.520115	-1.301937

C	5.303581	-0.092587	-1.151394
H	5.047689	-0.311479	2.241754
H	7.399818	-1.063962	1.975208
H	8.421441	-1.200520	-0.299457
H	7.064918	-0.583521	-2.300976
H	4.713880	0.167226	-2.033405
C	-4.292253	-0.461576	-0.619797
H	-3.127969	1.338156	-0.666341
H	-2.174060	-0.123751	-0.859999
C	-4.336981	-1.282937	-1.756373
C	-5.513991	-1.953723	-2.106796
C	-6.660012	-1.816306	-1.317310
C	-6.622620	-1.002874	-0.178369
C	-5.447978	-0.327453	0.166082
H	-3.443633	-1.391837	-2.378184
H	-5.532942	-2.587983	-2.995823
H	-7.578533	-2.342350	-1.586019
H	-7.513723	-0.891560	0.443522
H	-5.422148	0.302903	1.058636

58

1-all-*cis*-*C*$_2$ E(gas)=-1517.89874913 G(gas)=-1517.513603 E($CHCl_3$)=-1517.9213471

C	-1.486122	-1.030913	0.499876
C	-0.471727	-1.548792	1.287524
C	-2.667771	-0.180433	0.900276
N	0.397185	-2.110101	0.399913
H	-0.291405	-1.550825	2.357442
N	-0.063163	-1.971260	-0.874470
C	1.715377	-2.698388	0.636294
N	-1.202254	-1.322858	-0.806952
C	2.833267	-2.096305	-0.255181
H	1.940883	-2.595887	1.707592
H	1.691297	-3.766126	0.385204
O	3.429800	-2.852770	-1.022575
N	3.112787	-0.754521	-0.157878
C	2.643485	0.148602	0.899291
C	4.055498	-0.163103	-1.137343
C	1.483007	1.034067	0.520686
H	2.372391	-0.433479	1.791766
H	3.514151	0.760887	1.193337
C	0.378665	1.380158	1.278385
N	1.344543	1.607198	-0.716817
N	-0.393245	2.130367	0.444219
H	0.088484	1.164690	2.300532
N	0.209847	2.263079	-0.773675
C	-1.732506	2.671472	0.667140
C	-2.832754	2.070238	-0.248276
H	-1.969277	2.545910	1.733515
H	-1.723806	3.743280	0.435393
O	-3.403609	2.824832	-1.036276
N	-3.138563	0.733164	-0.143102
C	-4.075065	0.150580	-1.132467

H	-3.529346	-0.813667	1.175934
H	-2.415259	0.388772	1.807175
H	3.573705	0.734295	-1.552733
C	5.403575	0.177803	-0.532224
H	4.167241	-0.905708	-1.938189
C	5.824571	1.512962	-0.432071
C	7.072232	1.830409	0.119812
C	7.912484	0.812010	0.579175
C	7.501856	-0.524420	0.482016
C	6.257414	-0.839752	-0.069413
H	5.170370	2.309432	-0.798080
H	7.386894	2.874308	0.188393
H	8.887417	1.056157	1.007028
H	8.160333	-1.324097	0.829426
H	5.942316	-1.882401	-0.160879
H	-3.588328	-0.735788	-1.565723
C	-5.421821	-0.205952	-0.534283
H	-4.189478	0.905389	-1.921692
C	-5.850005	-1.541264	-0.478275
C	-7.098390	-1.870723	0.064921
C	-7.932088	-0.864160	0.560665
C	-7.514248	0.472553	0.508213
C	-6.269405	0.799525	-0.035188
H	-5.201807	-2.328836	-0.873298
H	-7.418623	-2.914626	0.098439
H	-8.907417	-1.117523	0.982099
H	-8.167497	1.263505	0.884156
H	-5.949898	1.843309	-0.092005

58

1-all-*cis*-S$_2$ E(gas)=-1517.90239297 G(gas)=-1517.517428 E($CHCl_3$)=-1517.92243075

C	1.529182	1.201915	-0.360741
C	0.728844	1.306742	0.764446
C	2.948969	0.750516	-0.548428
N	-0.475794	1.722161	0.282777
H	0.888769	1.070685	1.810382
N	-0.428631	1.892385	-1.067085
C	-1.731143	1.914582	1.019419
N	0.784344	1.568792	-1.452791
C	-2.956486	1.737688	0.093625
H	-1.726677	1.210021	1.863337
H	-1.783312	2.943172	1.400896
O	-3.519265	2.738366	-0.350607
N	-3.357710	0.453577	-0.201586
C	-2.949058	-0.749775	0.549423
C	-4.445280	0.283076	-1.187769
C	-1.529363	-1.201434	0.361640
H	-3.106305	-0.612404	1.631789
H	-3.650942	-1.543049	0.250518
C	-0.729094	-1.306175	-0.763606
N	0.475576	-1.721608	-0.282038
H	-0.889116	-1.070174	-1.809539

N	0.428509	-1.891889	1.067818
C	1.730889	-1.913801	-1.018794
N	-0.784450	-1.568327	1.453622
C	2.956294	-1.737088	-0.093070
H	1.783089	-2.942302	-1.400510
H	1.726333	-1.209043	-1.862546
O	3.519032	-2.737868	0.350995
N	3.357708	-0.453056	0.202234
C	4.445774	-0.282862	1.187880
H	3.106451	0.613593	-1.630818
H	3.650631	1.543797	-0.249035
H	-4.146028	-0.512062	-1.889905
C	-5.788858	-0.058185	-0.570392
H	-4.505801	1.229210	-1.742543
C	-6.417483	-1.280193	-0.853358
C	-7.660430	-1.594358	-0.289804
C	-8.286999	-0.685869	0.567822
C	-7.668445	0.538476	0.854376
C	-6.429981	0.851850	0.289201
H	-5.933148	-1.991715	-1.529099
H	-8.136802	-2.549712	-0.521216
H	-9.256015	-0.927657	1.009852
H	-8.159363	1.255811	1.516081
H	-5.955300	1.813420	0.499267
H	4.147182	0.512472	1.890071
H	4.506217	-1.228948	1.742756
C	5.789189	0.057796	0.569780
C	6.419381	1.278746	0.853807
C	7.662214	1.592248	0.289637
C	8.287059	0.684189	-0.569710
C	7.666903	-0.539084	-0.857359
C	6.428571	-0.851814	-0.291538
H	5.936339	1.989954	1.530801
H	8.139828	2.546783	0.521868
H	9.255961	0.925502	-1.012250
H	8.156454	-1.256098	-1.520425
H	5.952650	-1.812590	-0.502425

4.0 X-ray crystallography of cyclic tetraoligoamide 3

Crystals of **3** suitable for single crystal X-ray diffraction analysis were obtained by slow evaporation, dissolving 4 mg of the compound in acetonitrile. For the measurement, a colourless prismatic crystal of 0.22 mm x 0.07 mm x 0.04 mm was selected and mounted on a cryoloop with paratone oil.

Data collection was performed at room temperature with a Bruker D8 QUEST diffractometer equipped with a PHOTON detector using Cu*Kα* radiation (λ= 1.54178 Å).

Data indexing, integration and reduction were performed using CrysAlisPro ver. 1.171.42.51a.[7] Empirical absorption correction was performed with CrysAlisPro ver. 1.171.42.51a using spherical harmonics, implemented in SCALE3 ABSPACK scaling algorithm. The structure was solved using SHELXS[8] and refined through full matrix least-squares based on F^2 using the program SHELXL.[9] Non-hydrogen atoms were refined anisotropically, hydrogen atoms were positioned geometrically and included in structure factors calculations but not refined.

The side chain attached to the nitrogen atom N2 shows positional disorder with a refined occupancy of 0.669(7) and 0.331(7).

ORTEP diagram (Figure S13) were drawn using OLEX2.[10] In Table S2 are reported the crystallographic data.

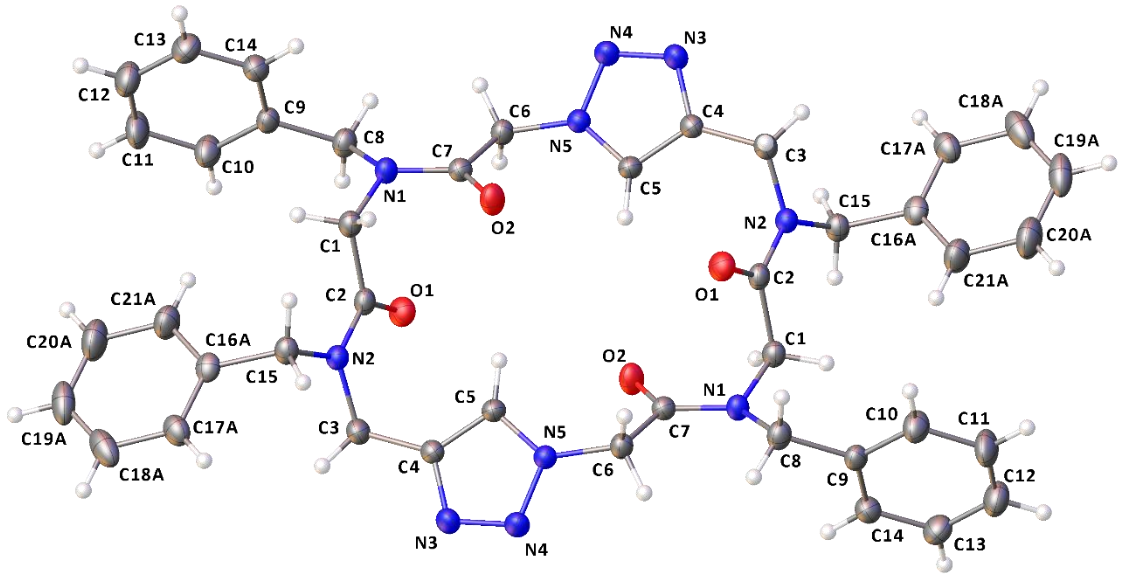

Figure S16. ORTEP drawing for compound **3**. Atom types: C grey, O red, N blue, H white. For clarity, only the atoms with the highest occupancy factor are shown. Ellipsoids are drawn at 20% probability level.

Table S2. Relevant crystallographic data for compound **3**.

	**3**
**T (K)**	296
**Formula**	$C_{42}H_{42}N_{10}O_4$
**Formula weight**	750.85
**System**	Monoclinic
**Space group**	$P2_1/n$
a **(Å)**	12.8093(6)
b **(Å)**	10.7877(4)
c **(Å)**	14.7191(6)
α **(°)**	90
β **(°)**	110.737(5)
γ **(°)**	90
V **(Å^3)**	1902.16(15)
**Z**	2
D_x **(g cm^{-3})**	1. 311
**λ (Å)**	1.54178
μ **(mm^{-1})**	0.710
F_{000}	792.0
**R1 (I > 2σI)**	0.0463(2425)
${}_{w}R_2$	0.1260(3718)
**N. of param.**	285
**GooF**	1.034
ρ_{min}, ρ_{max} **(eÅ^{-3})**	-0.12, 0.13

4.1 Intramolecular CO···CO interactions

Table S3. CO···CO distances and corresponding θ angles for compound **3.**

O1···C7 (d_1) (Å)	O2···C2 (d_2) (Å)	O1···C7-O2 (θ_1) (°)	O2···C2-O1 (θ_2) (°)
2.900(3)	3.058(3)	88.8(1)	81.5(1)

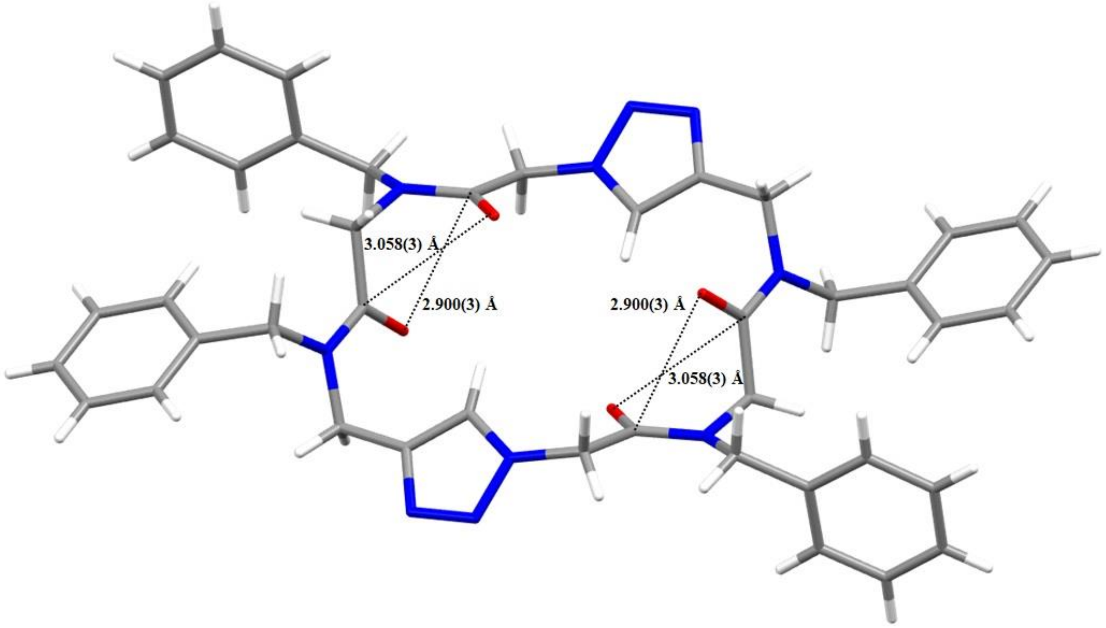

Figure S17. Reciprocal CO···CO interactions in the macrocycle, as shown by CO···CO distances below 3.22 Å. For clarity, only the atoms with the highest occupancy factor are shown.

5.0 References and notes

1) Althuon, D.; Rönicke, F.; Fürniss, D.; Quan, J.; Wellhöfer, I.; Jung, N.; Schepers, U.; Bräse, S. Org. Biomol. Chem., **2015**, *13*, 4226–4230.

2) Chang, Z.; Jing, X.; He, C.; Liu, X.; Duan,C. ACS Catal., 2018, 8, 1384–1391.

3) Gaussian 09, Revision A.02, Frisch, M. J.; Trucks, G. W.; Schlegel, H. B.; Scuseria, G. E.; Robb, M. A.; Cheeseman, J. R.; Scalmani, G.; Barone, V.; Mennucci, B.; Petersson, G. A.; Nakatsuji, H.; Caricato, M.; Li, X.; Hratchian, H. P.; Izmaylov, A. F.; Bloino, J.; Zheng, G.; Sonnenberg, J. L.; Hada, M.; Ehara, M.; Toyota, K.; Fukuda, R.; Hasegawa, J.; Ishida, M.; Nakajima, T.; Honda, Y.; Kitao, O.; Nakai, H.; Vreven, T.; Montgomery, J. A., Jr.; Peralta, J. E.; Ogliaro, F.; Bearpark, M.; Heyd, J. J.; Brothers, E.; N. Kudin, K.; Staroverov, V. N.; Kobayashi, R.; Normand, J.; Raghavachari, K.; Rendell, A.; Burant, J. C.; Iyengar, S. S.; Tomasi, J.; Cossi, M.; Rega, N.; Millam, J. M.; Klene, M.; Knox, J. E.; Cross, J. B.; Bakken, V.; Adamo, C.; Jaramillo, J.; Gomperts, R.; Stratmann, R. E.; Yazyev, O.; Austin, A. J.; Cammi, R.; Pomelli, C.; Ochterski, J. W.; Martin, R. L.; Morokuma, K.; Zakrzewski, V. G.; Voth, G. A.; Salvador, P.; Dannenberg, J. J.; Dapprich, S.; Daniels, A. D.; Farkas, O.; Foresman, J. B.; Ortiz, J. V.; Cioslowski, J.; Fox, D. J. Gaussian, Inc., Wallingford CT, **2009**

4) a) Becke, A. Phys. Rev. A 1988, 38, 3098– 3100. b) Perdew, J. P. Phys. Rev. B 1986, 33, 8822– 8824. c) Perdew, J. P. *Phys. Rev. B* **1986**, *34*, 7406– 7406.

5) Schaefer, A., Horn, H. and Ahlrichs, R. *J. Chem. Phys.* **1994**, *100*, 5829– 5835.

6) a) Barone, V. and Cossi, M. *J. Phys. Chem. A* **1998**, *102*, 1995– 2001. b) Tomasi, J. and Persico, M. *Chem. Rev.* **1994**, *94*, 2027– 2094.

7) Rigaku OD (2022). *CrysAlis PRO*, Version ver. 1.171.42.51a. Rigaku Oxford Diffraction.

8) G. M. Sheldrick, *Acta Cryst. A64*, **2008**, 112–122.

9) G. M. Sheldrick, *Acta Cryst. C71*, **2015**, 3–8.

10) O. V. Dolomanov, L. J. Bourhis, R. J. Gildea, J. A. K Howard and H. Puschmann, *J. Appl. Cryst.*, **2009**, *42*, 339–341.
